# Zic3 enables bimodal regulation of tyrosine hydroxylase expression in olfactory bulb and midbrain-derived neurons

**DOI:** 10.1038/s41420-025-02448-2

**Published:** 2025-04-11

**Authors:** Smitha Bhaskar, Jeevan Gowda, Akshay Hegde, Surya Chandra Rao Thumu, Shreetama Banerjee, Helen M Bellchambers, Narendrakumar Ramanan, Paloma Merchan Sala, Kenneth Campbell, Stephanie Ware, Jyothi Prasanna, Anujith Kumar

**Affiliations:** 1 Manipal Institute of Regenerative Medicine, Manipal Academy of Higher Education, Bangalore, India; 2 IFOM-inStem Joint Research Laboratory, Institute for Stem Cell Science and Regenerative Medicine (inStem), Bangalore, India; 3 School of Chemical and Biotechnology, SASTRA University, Thanjavur, India; 4 Centre for Neuroscience, Indian Institute of Science, Bengaluru, India; 5 Department of Medical and Molecular Genetics, Indiana University School of Medicine, Indianapolis, IN USA; 6 Division of Developmental Biology and Neurosurgery, Cincinnati Children’s Hospital Medical Center, University of Cincinnati College of Medicine, Cincinnati, OH USA

**Keywords:** Neuroscience, Cellular neuroscience

## Abstract

Tyrosine hydroxylase (TH) is the rate-limiting enzyme involved in the biosynthesis of catecholamines such as dopamine, norepinephrine, and epinephrine expressed in various regions of the brain, including the olfactory bulb (OB) and midbrain (MB). Previous studies demonstrated Zinc Finger transcription factor of the Cerebellum 3 (ZIC3) to regulate forebrain development, and *Zic1/Zic3* compound mutant mice displayed reduced OB size. However, the precise role of ZIC3 in TH regulation remains elusive. In this study, we attempted to understand the role of ZIC3 in TH regulation and its underlying mechanism. While loss of function of *Zic3* in OB-derived neurons led to down-regulation of TH expression, it could be rescued by over-expression of shRNA-resistant *Zic3*. Immunohistochemistry of OB of *Zic3* null mice showed a similar reduction in expression of TH. Promoter of TH lacks the consensus ZIC3 binding region, and mechanistic insights revealed ZIC3 to regulate TH expression by interacting with ER81, a known TH regulator. ZIC3 interaction with ER81 is indispensable for ER81 binding to the *Th* promoter, and it fine-tunes ER81-mediated *Th* regulation in OB. In MB, where TH levels are highest after birth, ZIC3 regulates TH expression both in vitro and in vivo. TH was significantly reduced in P0 *Zic3* null mice, as well as in *Zic3* shRNA stereotactically delivered in 7-month-old mice. Mechanistically, in the absence of ER81 in MB, ZIC3 chooses an alternative approach of binding to *Pitx3* promoter—a Dopaminergic (DA) fate determinant. Under the ectopic expression of ER81 in MB derived neurons, the propensity of ZIC3 binding to *Pitx3* promoter is compromised, and its occupancy on *Th* promoter encompassing ER81 binding site is established, finally culminating in desired TH expression. Together, these findings reveal a unique ZIC3-mediated bimodal regulation of TH in OB and MB derived neurons.

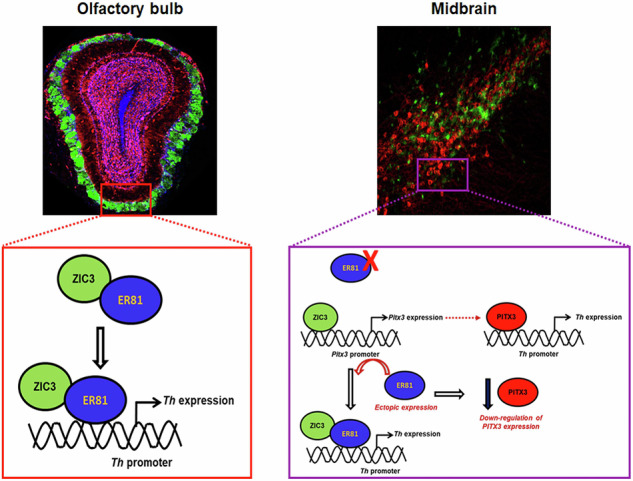

## Introduction

Neuronal diversity in the olfactory bulb (OB) is generated by a fairly defined group of molecular determinants in a spatiotemporal manner, thus ensuring neurochemical and morphological variations [1, 2]. The complex developmental programs determining the OB dopaminergic (DA) neurons are orchestrated by several transcription factors (TFs) [3]. Certain studies have appreciated the pivotal role of Zinc finger transcription factor in cerebellum 3 (ZIC3), belonging to the ZIC family TFs, in neural development and differentiation [4–6]. ZIC family comprises ZIC1-5 members [7, 8] and has domains similar to those of Gli proteins [9]. The *Zic3* gene is present on the X chromosome and is highly conserved between mice and humans [10, 11]. ZIC3 is abundantly expressed in ESCs and is shown to be essential for maintenance of pluripotency and self-renewal of ESCs and also in efficient derivation of iPSCs from mouse fibroblasts [12, 13]. In humans, the importance of ZIC3 is well reflected by its mutation leading to multiple disorders like heterotaxy (situs ambiguous) [14, 15], cerebellar anomaly [16, 17], and anorectal malformation [15].

ZIC family members are also known to play a crucial role in neuronal development [18]. ZIC family members are some of the earliest TFs to be expressed during gastrulation and are expressed earlier than that of proneural genes in the Neuroectoderm [4, 19]. Mice homozygous null for ZIC1 and ZIC3 have implications on expansion of tyrosine hydroxylase (TH) positive neurons and caused hypoplasia of the hippocampus, septum, and OB [6]. ZIC3 knockout mice exhibit the bent tail phenotype featuring the neural tube defects [20, 21]. A detailed expression profiling of ZIC3 in brain regions in recent years has reported its presence in the septal region and dorsal SVZ [22], thus suggesting its contribution to OB interneuron generation. Despite these insights, the role of ZIC3 in the regulation of TH-positive neuron generation is unclear.

In the present study, we report ZIC3 to be positively modulated upon exposing the animals to odor-enriched environment, and ZIC3 is expressed in a distinct micro-domain of OB neurons, where it positively regulates TH expression. Interestingly, ZIC3 deploys a distinct mechanism in OB and midbrain (MB) to fine-tune the expression of TH. In the absence of a direct binding site on the *Th* promoter, ZIC3 collaborates with ER81 in OB to drive the expression of *Th*, whereas in MB, ZIC3 indirectly dictates *Th* expression by occupying the *Pitx3* promoter. Thus, we propose ZIC3 to regulate *Th* expression in a bimodal fashion and in a spatially distinct manner.

## Results

### ZIC3 is essential for TH expression in OB-like neurons derived from mESCs

**Fig. 1:**
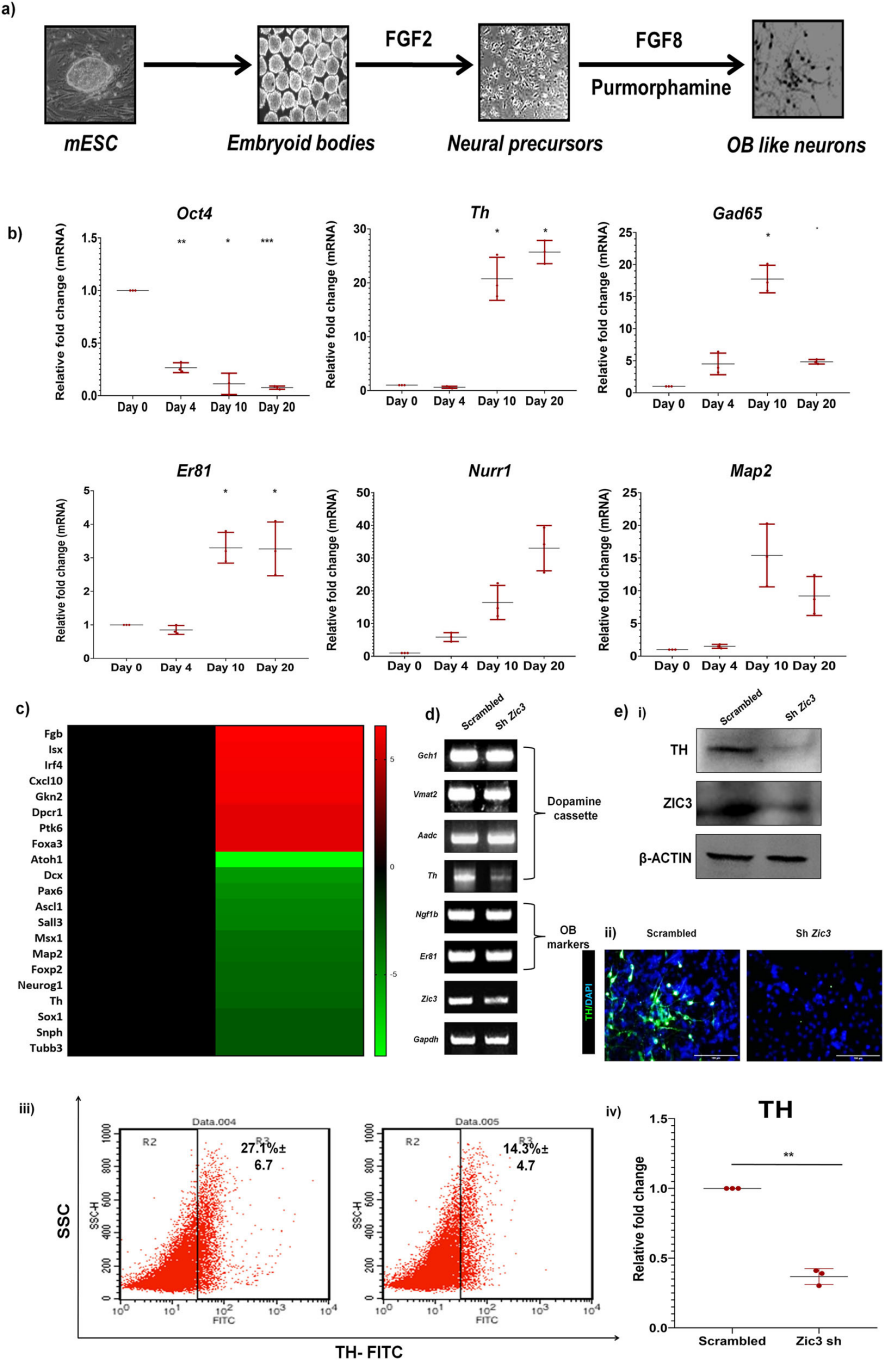
ZIC3 is essential for the generation of OB-like neurons from mESC. **a** Schematic of mESCs differentiation to OB neurons representing different stages and developmentally relevant cues, **b** transcript analysis of pluripotent marker *Oct4* and various OB neuronal markers during different days of differentiation, **c** microarray analysis of mESCs differentiated to neurons in presence and absence of *Zic3* represented as heat map, **d** transcript analysis showing specific reduction in *Th* expression upon *Zic3* knockdown in OB differentiation of mESCs, and **e** Western blot (i), immunofluorescence (ii) and flow cytometry (iii) analysis of TH expression and its quantification (iv) in OB like neurons derived from mESCs in presence and absence of *Zic3*. Scale bar represents 100 µm. Mean ± SE of biological triplicates (*n* = 3), ^*^p ≤ 0.05, ^**^p ≤ 0.01, ^***^p ≤ 0.001.

A study by Inoue et al. [6] showed the compound knockout of *Zic1* and *Zic3* to result in decreased size of the OB due to diminished neural progenitor expansion. However, an in-depth understanding of Zic-mediated OB development is unavailable to date. To determine the role of *Zic3* in OB differentiation, we differentiated mESCs to OB interneuron like cells after providing developmentally relevant cues (Fig. 1a). Characterization at the end of differentiation showed a gradual increase in OB genes *Th*, *Gad65*, *Er81*, and *Nurr1* and neuronal marker *Map2* with a concomitant down-regulation of pluripotency marker *Oct4* (Fig. 1b). These cells co-expressed TH with GAD65—which suggested the possibility of having OB DA like identity, and TUJ1 (Supplementary Fig. 1a) and stained positive for OB enriched transcription factors ER81 and NGF1β and neuronal marker MAP2 (Supplementary Fig. 1b). To understand any plausible implications of *Zic3* inhibition on the efficiency of this differentiation, we performed microarray analysis and found several neural markers like *Atoh1*, *Dcx*, *Pax6*, *Ascl1*, *Sall3* and specific DA marker *Th* down modulated in OB neurons lacking *Zic3* expression (Fig. 1c). Semi-quantitative PCR validated the downregulation of *Th* expression, but not the expression of other dopamine cassette genes *Aadc*, *Vmat2*, and *Gch1* (Fig. 1d) upon *Zic3* knock-down. Further, protein analysis using western blot (Fig. 1e-i), immunofluorescence (Fig. 1e-ii), and flow cytometry (Fig. 1e-iii) revealed ~50% reduction in TH expression (Fig. 1e-iv) upon *Zic3* knockdown. Thus, our study identified a novel role for ZIC3 in governing the expression of TH in OB DA-like neurons derived from mESCs.

### Expression analysis of ZIC3 and its association with odor perception in OB-neurons

**Fig. 2:**
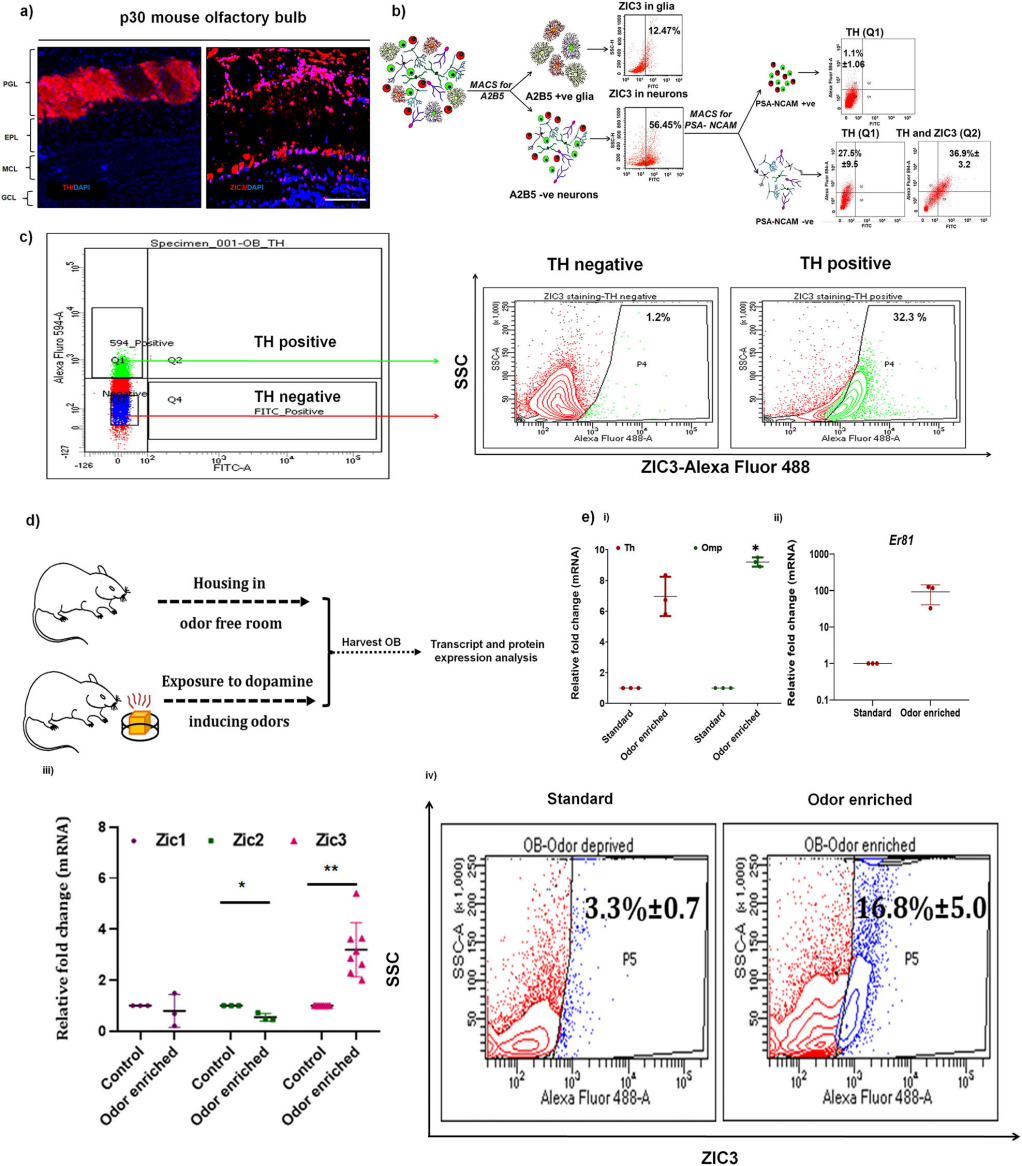
ZIC3 is present in a subset of OB neurons and is associated with odor perception. **a** Immunohistochemical staining for ZIC3 and TH representing their presence in the outermost periglomerular layer of mouse OB, **b** MACS to separate PSA-NCAM-ve and PSA-NCAM+ve cells from mouse OB and analysis for co-expression of TH and ZIC3, **c** FACS to fractionate TH+ve and TH-ve cells followed by flow cytometric analysis to score for co-expression of ZIC3 and TH. **d** Schematic representation of odor experiment and downstream gene and protein expression analysis. **e** (i) Transcript analysis of *Th*, *Omp* and, (ii) *Er81* in mice exposed to odor enriched environment in comparison to control condition, (iii) transcript analysis of *Zic* family members in OB of control and odor stimulated animals, (iv) ZIC3 protein analysis by flow cytometry in OB of mice exposed to odor and in comparison, to control. Scale bar represents 100 µm. Mean ± SE of biological triplicates (*n* = 3), ^*^p ≤ 0.05, ^**^p ≤ 0.01, ^***^p ≤ 0.001. All these experiments were performed in embryonic mouse OB-derived neurons cultured in vitro.

To corroborate the observation obtained from mESCs, as an initial experimental set up, we explored the expression domains of ZIC3 in various parts of the brain viz., cerebrum, cerebellum and OB and observed *Zic3* to be expressed in all the regions tested (Supplementary Fig. 2a). As our prime interest is in OB, we performed in situ hybridization (Supplementary Fig. 2b) and Immunohistochemical staining and observed ZIC3 expression in the outermost periglomerular layer primarily constituted by TH positive DA interneurons (Fig. 2a). To further corroborate this, A2B5 antibody stained positive glial cells were separated from A2B5 negative neuronal population in P2 mouse OB by MACS. About 56% of A2B5 negative neuronal cells expressed ZIC3 in comparison to 12% in glial cells (Fig. 2b). Neuronal cells were further labeled with PSA-NCAM antibody to fractionate neural progenitor cells from mature neurons. While 1% ± 1 of PSA-NCAM positive neural progenitors were TH positive, about 27.5% ± 9.5 of PSA-NCAM negative mature neurons expressed TH. Amongst PSA-NCAM negative mature neurons, 36.9% ± 3.2 of the cells were both ZIC3 and TH positive, thus establishing the presence of a subset of OB neurons that are ZIC3 positive. In an independent approach, we used FACS to segregate TH-positive and negative neurons, wherein 20% of the cells were positively labeled for TH. Staining of fractionated TH-positive cells showed 32.3% of TH-positive cells to co-express ZIC3 (Fig. 2c), reiterating the presence of ZIC3 in a subset of OB neurons.

Exposure of animals to natural odor stimulants activates OB glomeruli and other dopamine-producing brain regions [23, 24]. To see if ZIC3 exhibits any modulation in response to odor enrichment, we exposed mice to various natural odorants (Fig. 2d). Transcript analysis at the end of odor enrichment regimen exhibited up-regulation in the levels of *Th* by ~6.5-fold, *Omp* by ~8-fold (Fig. 2e-i) along with *Er81* by ~90-fold (Fig. 2e-ii) in animals exposed to odorants compared to control mice. We screened for the expression of *Zic* family members in OB upon odor stimulation and found up-regulation of *Zic3*, but not *Zic1* and *Zic2* (Fig. 2e-iii). Protein analysis showed a significant 9% increase in TH expressing cells (Supplementary Fig. 2b-i, ii) with a concomitant 12.5% increase in ZIC3 positive cells (Fig. 2e-iv) in animals exposed to odor compared to the control group. Altogether, our results so far suggest that ZIC3 is expressed in a subset of OB neurons and positively responds to odor stimulation.

### ZIC3 is essential for the generation and maintenance of TH+ve OB-neurons

**Fig. 3:**
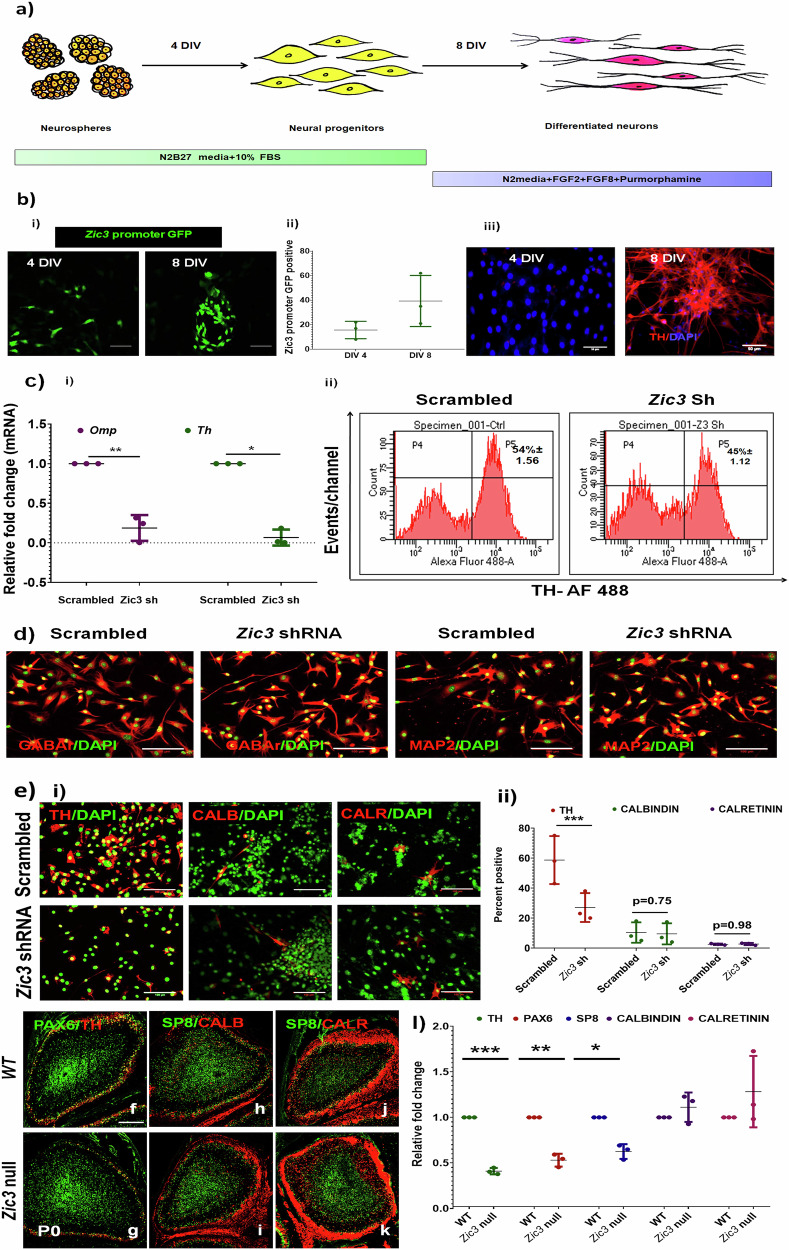
ZIC3 regulates TH expression in OB-derived interneurons. **a** Schematic diagram representing the differentiation of OB neural progenitors to TH-positive neurons. **b** (i) Expression of *Zic3* during the course of differentiation using *Zic3*-promoter GFP construct transfected in OB derived neurons, (ii) quantification of GFP expression and (iii) TH expression analysis by immunofluorescence at 4 DIV and 8 DIV. **c** (i) Transcript analysis of *Omp* and *Th* and (ii) TH protein analysis by flow cytometry in OB derived neurons transduced with either scrambled or *Zic3* shRNA, **d** Immunofluorescence staining of GABAr and MAP2 in cells transduced with either *Zic3* shRNA or scrambled control, and **e** (i) Immunofluorescence analysis of TH, CALBINDIN and CALRETININ in cells transduced with either *Zic3* shRNA or scrambled control and (ii) quantification of the immunofluorescent positive cells. **f**–**k** Images showing the expression of interneuron markers TH and PAX6 (**f**, **g**); SP8 and CALBINDIN (**h**–**i**), and SP8 and CALRETININ (**j**, **k**) in WT and *Zic3* null P0 mouse OB, **l** quantification of OB interneurons in WT and *Zic3* null P0 mice. Scale bar represents 100 µm. Mean ± SE of three independent biological triplicates (*n* = 3), ^*^p ≤ 0.05, ^**^p ≤ 0.01, ^***^p ≤ 0.001. *n* = 5 in case of (**f**–**k**). All these experiments were performed in mouse OB-derived neurons in culture and in vivo.

To understand the role of ZIC3 in OB interneuron differentiation, we chose embryonic mouse OB (E12.0–E14.0) derived neurons. Differentiated OB neurons (Fig. 3a) were characterized by the co-expression of GABA receptor B (GABA recB) with TH, expression of CALBINDIN (CALB), CALRETININ (CALR), and ER81. Absence of a MB DA specific marker PITX3 reassured the exclusive OB identity of these cells (Supplementary Fig. 3a). Upon culturing the OB progenitors as neurospheres followed by monolayer culture, the cells expressed progenitor markers SOX1 and NESTIN on 4th day of in vitro differentiation (DIV) (Supplementary Fig. 3b, c) and TUJ1 and TH with co-expression of GABA rec1B—a hallmark of OB DA neurons, upon 8th DIV (Supplementary Fig. 3c). Using *Zic3*-promoter tagged to GFP, we observed an increased induction of *Zic3* expression during the course of differentiation (Fig. 3b-i) with 92.9% higher *Zic3* promoter-GFP+ve cells at the end of differentiation (Fig. 3b-ii). The expression pattern correlated well with that of TH protein expression (Fig. 3b-iii).

To understand the role of *Zic3* in OB-neuronal differentiation, we performed shRNA-mediated knockdown of *Zic3* and validated the efficiency of inhibition by observing the downregulation of *Zic3* and its downstream target, NANOG protein in mESCs (Supplementary Fig. 3d, e). OB-derived neurons with *Zic3* knockdown failed to express *Th* transcript in comparison to scrambled control (Fig. 3c-i), and a decrease in TH+ve cells was observed (Fig. 3c-ii). However, the mature neuronal genes MAP2 and GABAergic marker remained unaltered upon *Zic3* inhibition (Fig. 3d). *Zic3* knockdown also did not show any evident effect on OB CALB and CALR +ve interneurons (Fig. 3e-i, ii). Inhibition of another ZIC family member,*Zic1*, failed to cause any significant modulation in *Th* expression (Supplementary Fig. 3f-i, ii), advocating a specific role for *Zic3* in TH regulation.

Though our results so far suggested the expression of ZIC3 in OB interneurons and its role in regulating TH expression, its contribution to the generation of OB interneurons in vivo remained to be investigated. To fulfill this, we obtained WT and *Zic3* null mice at P0, and the brains were fixed and sectioned. Further, we stained the sections for pan interneuron marker SP8 (Fig. 3h, i) and found a 40% decrease (Fig. 3l) in SP8+ population. Upon staining for specific interneuron markers-TH (Fig. 3f, g), CALBINDIN (CALB) (Fig. 3h, i) and CALRETININ (CALR) (Fig. 3j, k), we found a significant 57% (Fig. 3l) reduction in TH+ve DA neurons. Interestingly, we observed an approximate 50% reduction in the PAX6 +ve interneurons in *Zic3* null mice (Fig. 3f). However, any modulation in CALR and CALB +ve in *Zic3* null mice OB with respect to WT was not evident. In all, the *Zic3* null model displayed a significant down-regulation in DA interneurons of OB.

### Zic3 positively regulates TH expression in OB-derived neurons

**Fig. 4:**
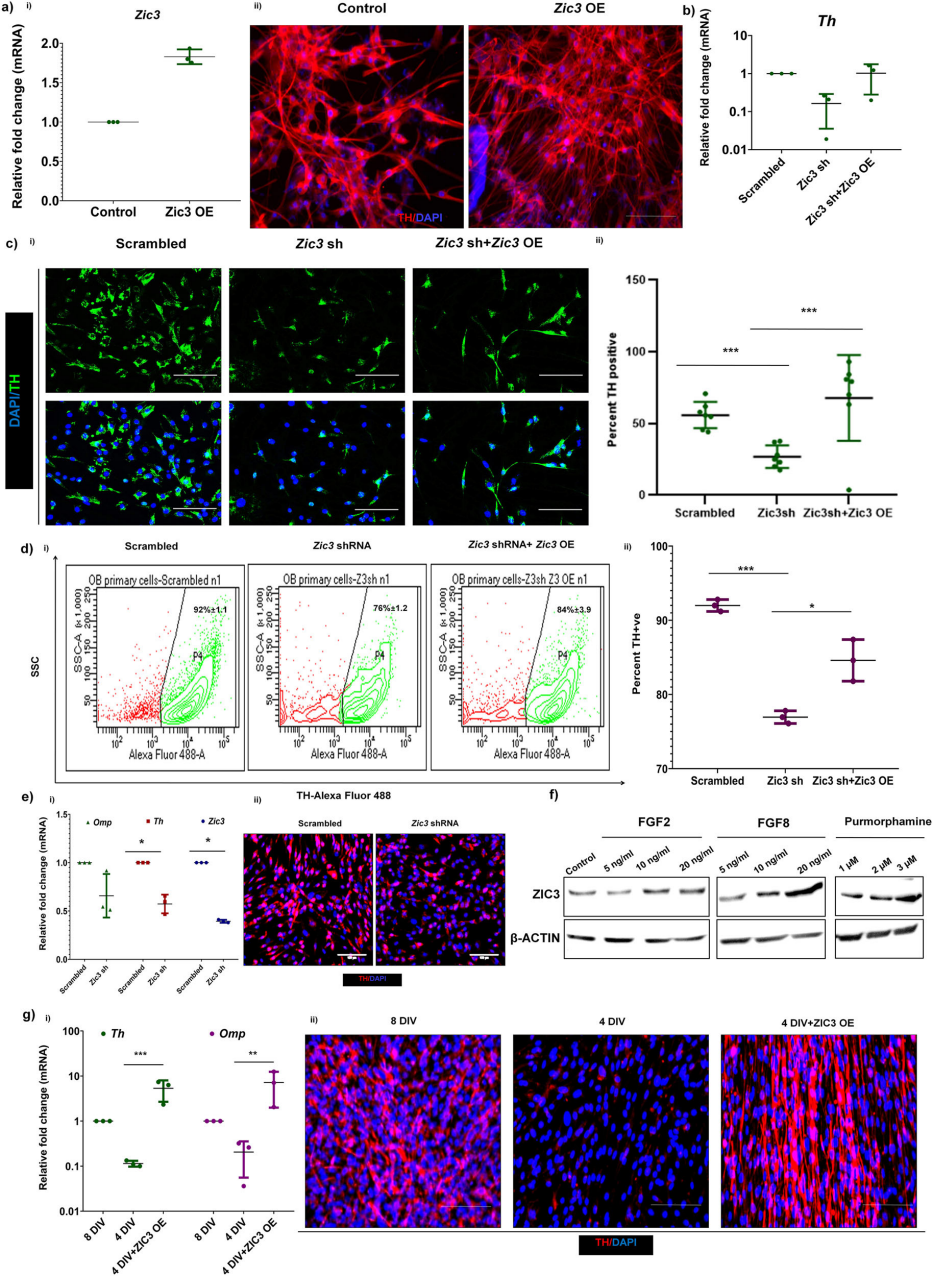
Overexpression of ZIC3 is sufficient to enhance TH expression in OB-derived neural progenitors. **a** (i) Transcript and (ii) protein analysis of TH in cells overexpressing *Zic3*. **b** Transcript analysis of *Th* in OB primary neurons with *Zic3* shRNA and rescue in *Th* expression by simultaneous overexpression of *Zic3*, **c** Immunofluorescence staining for TH (i) and its quantification (ii) in OB derived neurons cultured in absence of *Zic3* and simultaneous overexpression of *Zic3* in shRNA condition, **d** expression of TH (i) and its quantification (ii) upon *Zic3* inhibition and simultaneous over-expression as analyzed by flow cytometry, **e** mRNA (i) and protein expression (ii) analysis of TH in OB derived neurons with *Zic3* inhibition post differentiation, **f** expression analysis of ZIC3 in presence of cytokines FGF2, FG8 and Purmorphamine that are a part of the differentiation cocktail to differentiate OB progenitor to DA-like neurons, and **g** transcript (i) and protein (ii) analysis of TH expression in OB cells differentiated in presence and absence of differentiation cues and over-expression of *Zic3* in undifferentiated cells. Scale bar represents 100 µm. Mean ± SE of three independent biological triplicates (*n* = 3), ^*^p ≤ 0.05, ^**^p ≤ 0.01, ^***^p ≤ 0.001.

Overexpression of *Zic3* enhanced TH expression (Fig. 4a-i, ii) and reconstituting *Zic3* overexpression in cells harboring *Zic3* shRNA restored *Th* expression (Fig. 4b). Immunostaining for TH revealed approximately 52% reduction in TH +ve cells in *Zic3* sh condition in comparison to the scrambled control, which was rescued upon *Zic3* over-expression (Fig. 4c-i, ii). Flow cytometry showed TH expression in 92% ± 1.1 of the scrambled control cells, which was reduced to 76% ± 1.2 with *Zic3* shRNA, and the TH expression was rescued to 84% ± 3.9 cells upon *Zic3* overexpression (Fig. 4d-i, ii). The knockdown of *Zic3* in terminally differentiated neurons also showed drastic down-regulation of both *Th* and *Omp* mRNA (Fig. 4e-i) and TH protein (Fig. 4e-ii), inferring ZIC3 participates in the maintenance of TH in mature DA neurons also. Individual cytokines FGF2, FGF8 and Purmorphamine used in our OB primary neuron differentiation protocol, all in a dose dependent manner increased ZIC3 expression (Fig. 4f). In order to see if ZIC3 could compensate for the absence of these differentiation inducers, we over-expressed ZIC3 in cells cultured without any differentiation cues and found enhanced expression of TH (Fig. 4g-i, ii), indicating ZIC3 to be sufficient to drive the expression of TH in neural progenitors.

### ZIC3 and ER81 co-regulate TH expression in OB-neurons

**Fig. 5:**
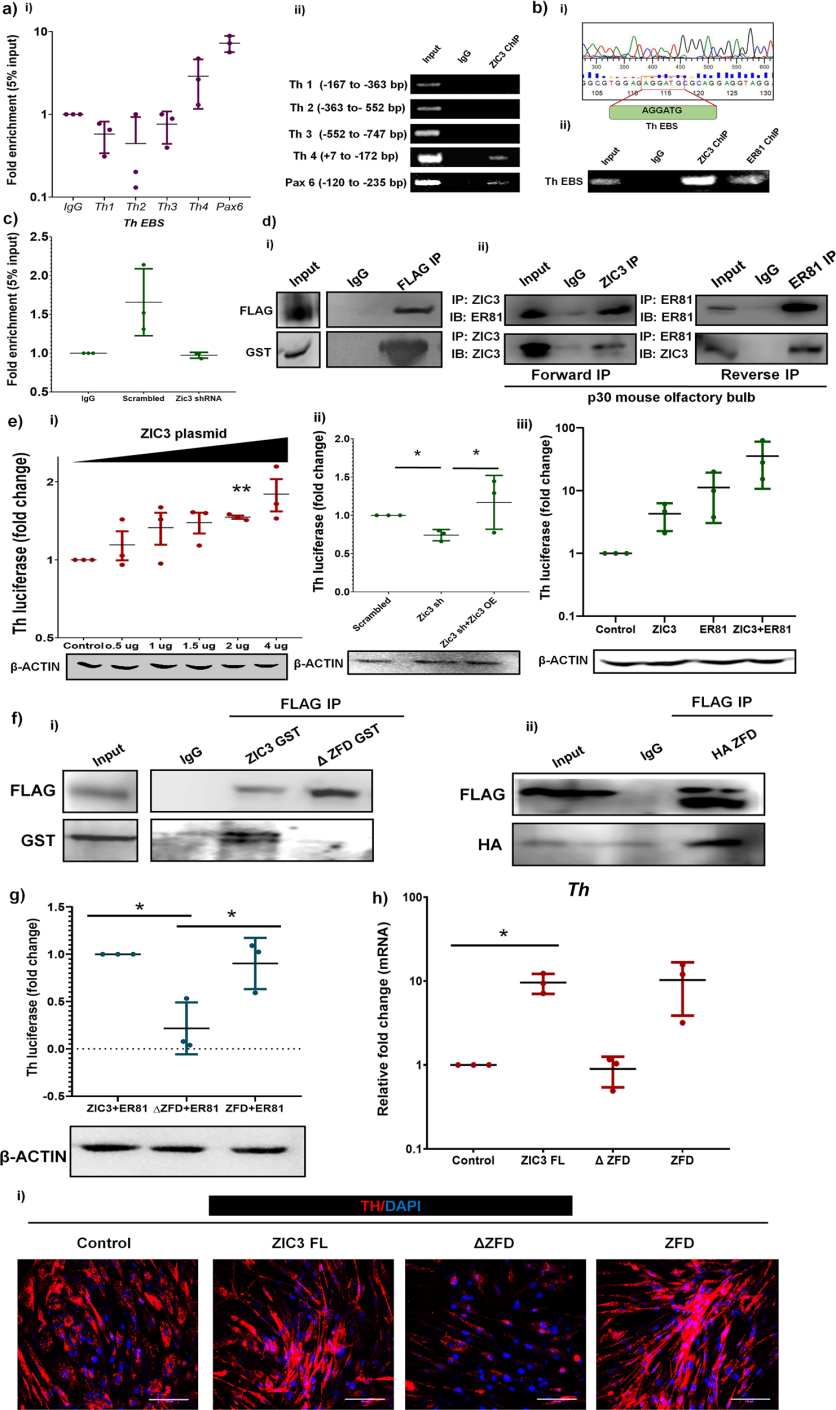
ZIC3 and ER81 interact with each other to regulate TH expression in mouse OB. **a** ChIP analysis performed using mouse OB tissue lysate showing ZIC3 occupancy on various regions of mouse *Th* promoter using region-specific primers by (i) qPCR and (ii) conventional PCR. *Pax6* was used as a positive control for ZIC3 binding. **b** Sanger sequencing result of ChIPed DNA (i) and ChIP validation (ii) showing binding of ZIC3 and ER81 to *Th* promoter region in mouse OB, **c** ChIP analysis of ER81 binding to *Th* promoter in presence and absence of *Zic3* in OB primary neuron derived cultures in vitro*.* **d** (i) Co-immunoprecipitation analysis in HEK-293T cells overexpressing immunoreactive tags FLAG-ER81 and ZIC3-GST. Immunoprecipitation was performed using FLAG antibody and immunoblot performed using GST antibody, (ii) endogenous protein interaction between ZIC3 and ER81 was confirmed in mouse OB tissue by forward immunoprecipitation using ZIC3 primary antibody for immunoprecipitation and immunoblot using ER81 primary antibody and reverse immunoprecipitation using ER81 primary antibody for immunoprecipitation and immunoblot using ZIC3 primary antibody, **e** (i) analysis of activation of *Th* promoter-luciferase in presence of increasing concentration of *Zic3* plasmid, Western blotting of β-actin used as loading control, (ii) *Th* – promoter analysis by luciferase in cells expressing *Zic3* shRNA and simultaneous over-expression of *Zic3* in *Zic3* shRNA condition, β-actin used as loading control and (iii) *Th*-promoter analysis by luciferase in individual and in combined over-expression of ZIC3 and ER81 conditions. Western blots depict the overexpression of ZIC3 and ER81 in their respective conditions, and β-actin was used as a loading control. **f** Co-immunoprecipitation analysis showing interaction between ZIC3-FL and ER81 but not the ZFD deletion mutant (ΔZFD) (i), and overexpression of the Zinc finger domain (ZFD) showed it to be sufficient to interact with ER81 (ii). Immunoprecipitation was performed using the FLAG antibody, and an immunoblot was performed using the GST antibody. **g** Analysis of activation of *Th* promoter-luciferase upon ectopic expression of ZIC3-FL, ΔZFD, and ZFD along with FLAG-ER81, β-actin used as loading control. **h** Transcript and, **i** protein expression of TH in OB-derived neurons over-expressing ZIC3-FL, ΔZFD, and ZFD plasmids. Scale bar represents 100 µm. Mean ± SE of three independent biological triplicates (*n* = 3), ^*^p ≤ 0.05, ^**^p ≤ 0.01, ^***^p ≤ 0.001. The *Th* 4 is referred to as *Th* EBS (EBS).

ZIC3 is traditionally viewed as a distal regulator and is a transcriptional activator [25]. To understand the mechanism behind ZIC3-mediated regulation of *Th*, we performed an *in-silico* search for the ZIC3 consensus binding site on the *Th* regulatory region. Though up to −3 kb, no consensus binding site for ZIC3 on mouse *Th* promoter was found, ChIP-PCR analysis for different regions of *Th* promoter with tiling primers identified the occupancy of ZIC3 encompassing +7 bp to −172 bp region on *Th* promoter (Fig. 5a-i, ii). Sequencing the promoter region occupied by ZIC3 showed the presence of a binding site of ETS family TF ER81 (Fig. 5b-i)-previously implicated in positive regulation of *Th* expression in mouse OB [26]. We henceforth refer to this as the ER81 binding site (EBS). Genetic knockdown of *Er81* (Supplementary Fig. 4a-i) showed a reduction in TH expression (Supplementary Fig. 4a-ii, b), supporting the previous study [26]. The occupancy of ER81 on *Th* promoter was re-confirmed by ChIP-qPCR, which showed an enrichment in the same region as that of occupancy of ZIC3 (Fig. 5b-ii). Further, the inhibition of *Zic3* in differentiated OB-derived neurons led to a compromised binding of ER81 to *Th* promoter (Fig. 5c). Co-immunoprecipitation assay revealed a direct interaction between GST-ZIC3 and FLAG-ER81 (Fig. 5d-i). In addition, we also performed co-immunoprecipitation (forward and reverse) using mouse OB tissue, which also showed similar interaction between ZIC3 and ER81 (Fig. 5d-ii). This interaction was true for ZIC3, but not ZIC1 (Supplementary Fig. 4c), suggesting the specificity. Functional analysis using *Th*-luciferase assay showed a gradual increase in its expression with increasing concentration of *Zic3* plasmid (Fig. 5e-i). Inhibition of *Zic3* reduced *Th*-luciferase by 30%, which was rescued by *Zic3* overexpression (Fig. 5e-ii). We over-expressed ZIC3, ER81 individually and in combination, and observed the highest activation of *Th*-promoter when both the TFs were combined (Fig. 5e-iii). This result suggested the ZIC3-mediated TH regulation to be via its interaction with ER81.

ZIC3 consists of five highly conserved zinc finger domains (ZFD 1–5), and studies have advocated the involvement of ZFD in nuclear localization [27], regulation of ZIC3 protein stability [28], and protein-protein interaction [29]. While the deletion mutants of ZIC3-ΔZF5, ΔZF4–5, ΔZF3–5 were able to localize to the nucleus, ΔZF2–5 and ΔZFD accumulated in the cytoplasm due to loss of nuclear localization signal (NLS) (Supplementary Fig. 5). Interaction studies showed only ZIC3-FL but none of the zinc finger mutants could interact with ER81 (Fig. 5f-i). Interestingly, just the ZFD was sufficient to physically associate with ER81 (Fig. 5f-ii). ZIC3 lacking ZFD failed to drive the *Th*-luciferase due to a lack of its interaction with ER81. However, substitution with ZFD protein along with FLAG ER81 was sufficient to prompt *Th*-luciferase expression to the extent of that by ZIC3 FL and ER81 together (Fig. 5g). To understand the functional role of interaction domains, we overexpressed ZIC3-FL, ΔZFD and ZFD and observed that only full length ZIC3-GST and ZFD-GST clones were able to up-regulate TH expression during OB-neuron differentiation but not ΔZFD lacking the functional domains (Fig. 5h, i).

A deeper examination of ZIC3-ER81 crosstalk showed that in the absence of ZIC3, over-expression of ER81 rescued the expression of TH (Supplementary Fig. 6a, b) to the level of scrambled control. Over-expression of ER81 enhanced the expression of *Zic3* even under the influence of *Zic3* shRNA (Supplementary Fig. 6c), thus enabling the retention of *Th* expression. ChIP analysis showed the direct binding of ER81 on *Zic3* promoter (Supplementary Fig. 6d), thus satisfactorily explaining ER81-mediated rescue of TH expression upon *Zic3* inhibition. On the other hand, supplementing *Zic3* in cells lacking *Er81* could not elicit a rescue of *Th* expression (Supplementary Fig. 6e). Thus, all these data together established a novel ZIC3-ER81-*Th* axis in OB TH-positive neurons.

### ZIC3 regulates TH expression in MB-neurons

**Fig. 6:**
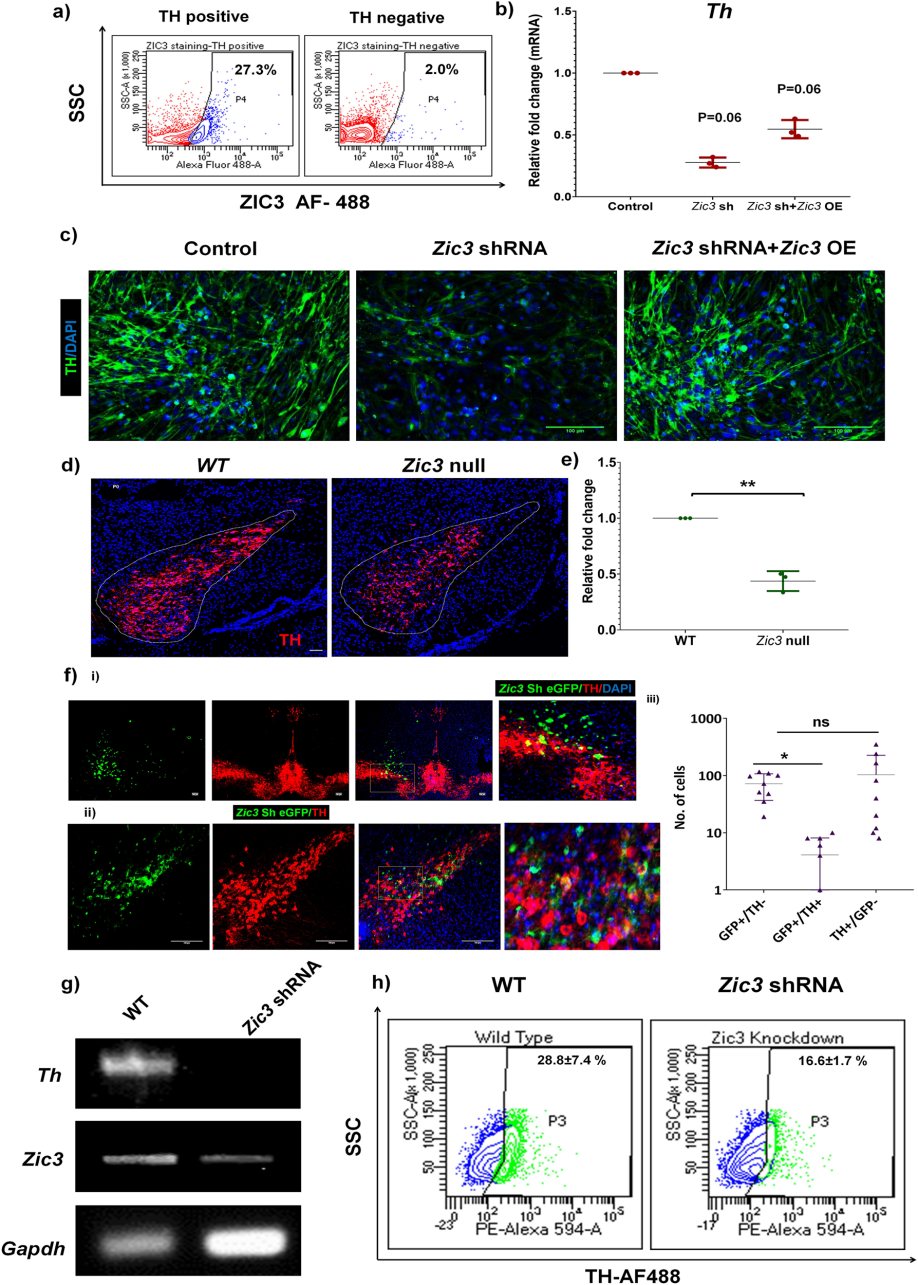
ZIC3 positively regulates TH expression in MB-neurons. **a** Flow cytometric analysis of ZIC3 expression in TH positive and negative cells sorted from mouse MB, **b** transcript and **c** protein analysis of TH in cells expressing *Zic3* shRNA and simultaneous over-expression of *Zic3*. Expression (**d**) and quantification (**e**) of TH+ve neurons in WT and *Zic3* null P0 mice MB, and **f** (i) in vivo expression analysis of TH in mouse MB upon stereotactic injection of *Zic3* shRNA-eGFP (ii) its higher magnification and (iii) quantification of TH+/GFP−, TH+/GFP+ and TH−/GFP+ cells. **g** Transcript and, **h** flow cytometry analysis of TH in cells obtained from mouse MB cells injected with *Zic3* shRNA. Scale bar represents 100 µm. Mean ± SE of three independent biological triplicates (*n* = 3), ^*^p ≤ 0.05, ^**^p ≤ 0.01, ^***^p ≤ 0.001. *n* = 5 in case of (**d**, **e**).

Alongside OB, another major source of TH-positive cells is the MB consisting of ventral tegmental area and substantia nigra. To test the role of ZIC3 in MB-neurons, we sorted TH positive neurons from 4-week-old mouse MB and stained for ZIC3, wherein ~27% of the cells co-expressed TH and ZIC3 (Fig. 6a). Neural progenitors derived from E12.0 to E14.0 mouse MB primary culture were positive for SOX1 and NESTIN and their differentiated counterparts expressed TUJ1 and TH (Supplementary Fig. 7a). Reduction in *Zic3* expression by shRNA (Supplementary Fig. 7b-i, ii) resulted in 50% reduction of *Th* mRNA with respect to scrambled control which was partially restored upon *Zic3* over-expression (Fig. 6b). Immunofluorescence staining showed an evident restoration of TH expression in cells over-expressing *Zic3* in comparison to *Zic3* shRNA (Fig. 6c).

In order to test our observations in vivo, we stained WT and *Zic3* null P0 mouse MB sections for TH. Upon quantification, we found about 60% reduction in TH +ve cells in *Zic3* null brain samples, thus advocating the effect of ZIC3 on TH expression to hold true in vivo (Fig. 6d, e). To see if *Zic3* has any implications on maintenance of TH expression in these MB neurons, we performed a stereotactic delivery of *Zic3* shRNA tagged to eGFP in 7 months old mice MB region and found that 56% ± 7.8 cells lacking *Zic3* were GFP+ve and TH-ve and 1.2% ± 0.3 of cells were GFP+ve and TH+ve (Fig. 6f-i, ii). Further, transcript analysis showed a drastic reduction in *Th* expression in the mice injected with *Zic3* shRNA (Fig. 6g). Flow cytometric staining showed a ~ 12% reduction in the TH+ve cells in *Zic3* shRNA injected mice (Fig. 6h). This result demonstrated ZIC3 to act as a positive regulator of TH in mouse MB, similar to our observations in OB.

### ZIC3 switches molecular partners in OB and MB neurons to regulate TH expression

**Fig. 7:**
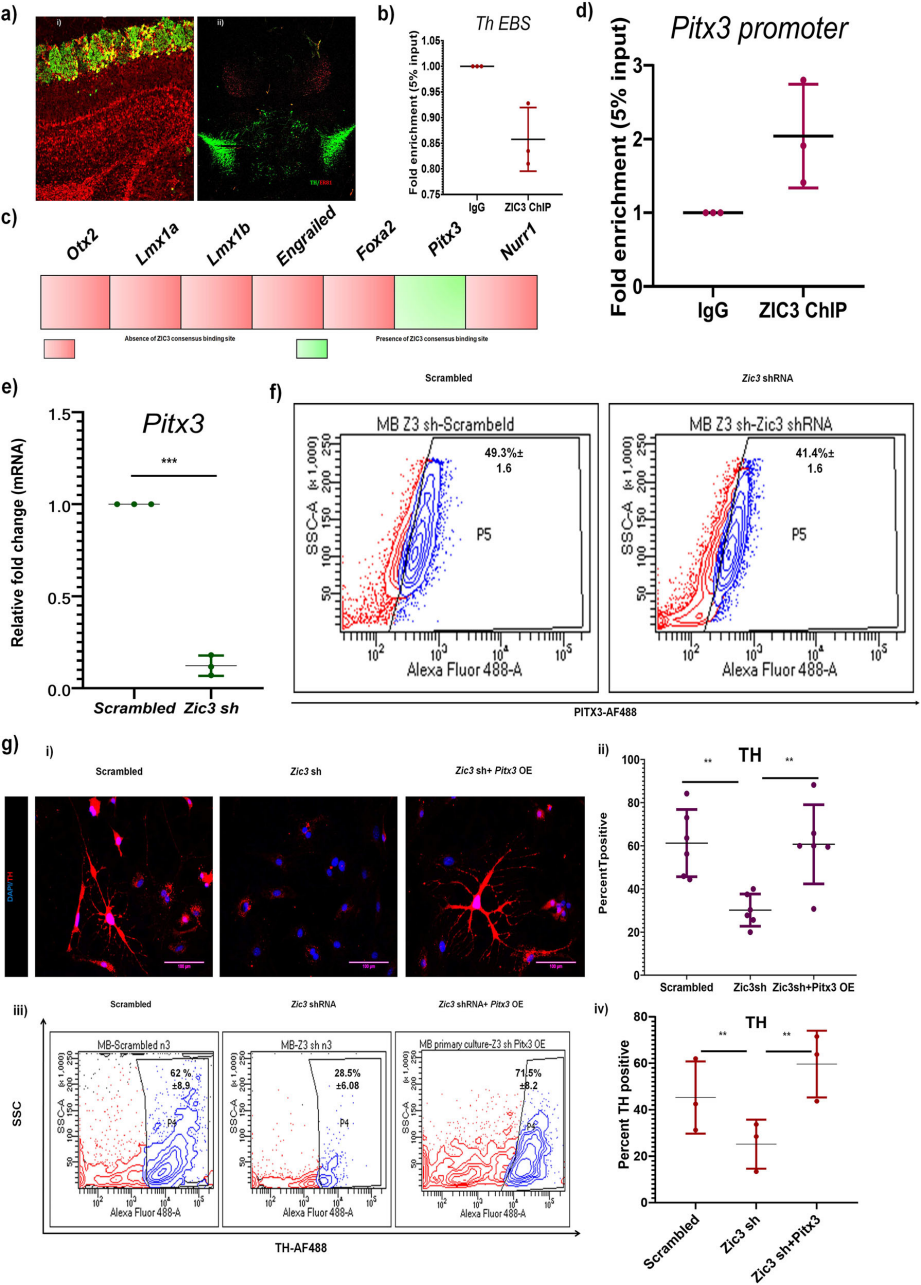
ZIC3 differentially regulates TH in mouse OB and MB by switching between ER81 and PITX3. **a** Immunohistochemistry of OB and MB tissue showing the presence of ER81 in OB but not in MB, **b** ChIP analysis showing absence of ZIC3 binding on *Th* promoter region where ER81 binds, **c** schematic representation of key MB specific gene promoters with presence and absence of ZIC3 consensus binding site in the region of −3000 bp from the transcript start site, and **d** ChIP analysis showing occupancy of ZIC3 on *Pitx3* promoter in mouse MB tissue lysate as analyzed by qPCR. **e** Reduction in *Pitx3* mRNA and, **f** PITX3 protein expression as analyzed by flowcytometry upon *Zic3* inhibition in MB derived neurons. **g** Immunofluorescence for TH (i), its quantification (ii), flow cytometric analysis (iii), and its quantification (iv) of TH-positive cells in MB-neurons upon *Zic3* inhibition along with *Pitx3* over-expression. Scale bar represents 100 µm. Mean ± SE of three independent biological triplicates (*n* = 3), ^*^p < 0.05, ^**^p < 0.01, ^***^p < 0.001. All these experiments [except (**a**)] were performed in embryonic mouse brain-derived cultures in vitro.

We were intrigued to know whether ZIC3 adopts a similar mechanism in MB as observed in OB to regulate TH expression. However, the MB region lacks the expression of ER81 (Fig. 7a–i, ii)—the interacting partner of ZIC3 as found in OB. This corroborates well with the literature that showed expression of ER81 to be absent in MB-neurons [30]. ChIP analysis in MB tissue lysate showed a lack of ZIC3 binding to the *Th* promoter region, potentially due to the absence of its interacting partner—ER81 (Fig. 7b). This suggested a possible alternate mechanism that could be adopted by ZIC3 in MB to regulate the expression of TH. Amongst in silico promoter analysis of different DA regulators up to −3 kb, only the promoter region of *Pitx3*—the penultimate marker of MB DA neurons harbored a ZIC3 binding site (Fig. 7c). We performed ChIP assay in 4-weeks-old mouse MB lysate and found approximately 2-fold enrichment in ZIC3 binding to *Pitx3* promoter (Fig. 7d). Also, the inhibition of *Zic3* in MB derived neurons led to significant down-regulation of PITX3 expression (Fig. 7e, f). While knockdown of *Zic3* abrogates *Th* expression in MB derived neurons, over-expression of *Pitx3* along with *Zic3* shRNA retains TH expression (Fig. 7g-i, ii). Flow cytometry also reiterated the maintenance of TH expression in *Pitx3* overexpressing cells subjected to *Zic3* loss of function (Fig. 7g-iii, iv). This result conclusively established *Pitx3* as the downstream target of ZIC3 in MB and a novel ZIC3-PITX3-TH molecular axis operating in MB TH-positive cells.

**Fig. 8:**
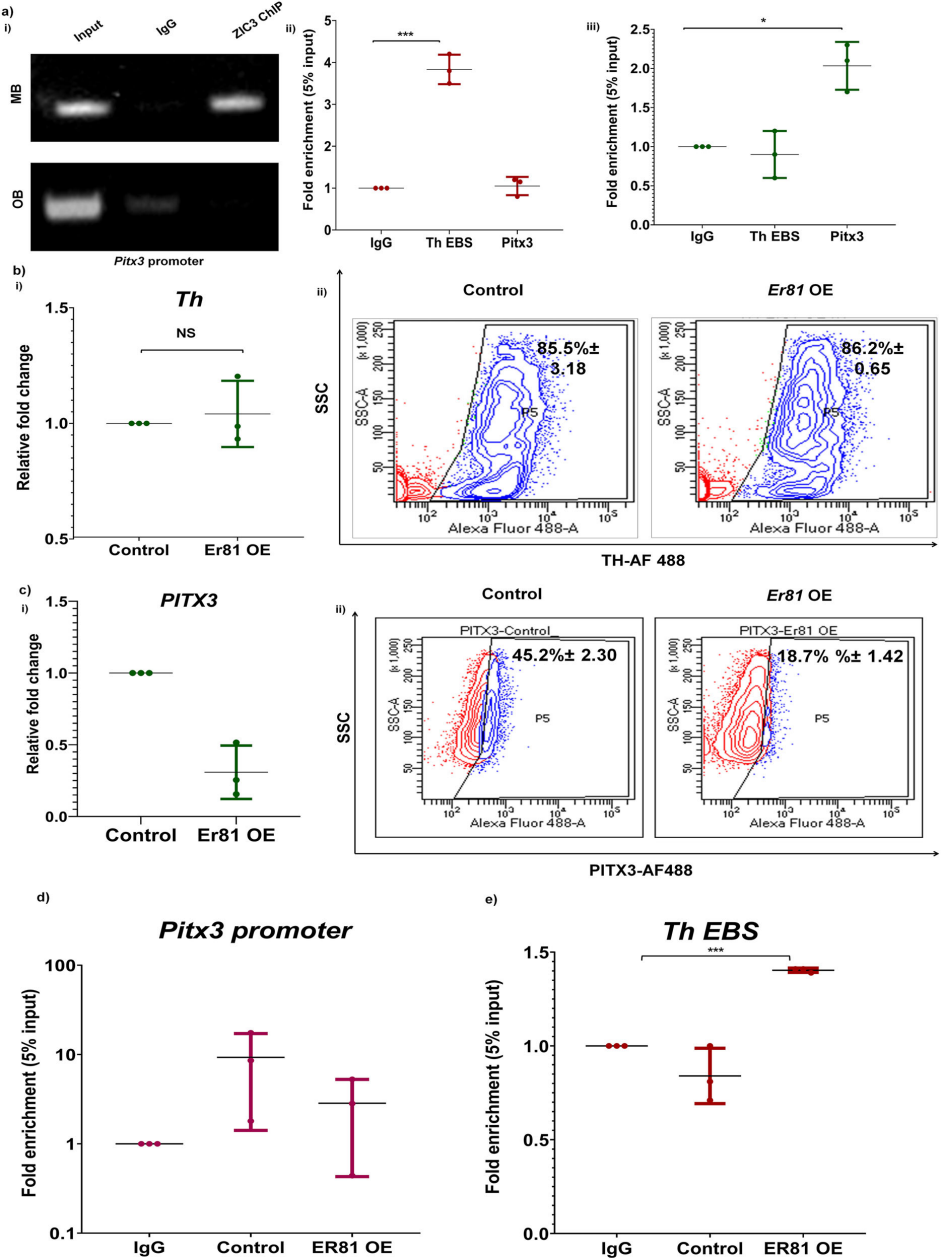
Overexpression of ER81 in MB cells hijacks ZIC3 from Pitx3 promoter and enhances its binding on EBS on *Th* promoter. **a** (i) ChIP analysis using ZIC3 primary antibody showing ZIC3 binding to *Pitx3* promoter specifically in mouse MB but not OB as analyzed by conventional PCR, (ii) ChIP analysis showing non-occupancy of ZIC3 on *Pitx3* promoter in mouse OB tissue lysate and specifically occupying *Pitx3* promoter in MB and (iii) as analyzed by qPCR. **b** (i) Transcript and (ii) protein expression analysis of TH in the presence and absence of ER81 overexpression condition in MB cells. **c** (i) Transcript and (ii) protein expression analysis of PITX3 in MB derived neurons over-expressing ER81. **d** ChIP analysis showing decreased occupancy of ZIC3 on *Pitx3* promoter in MB tissue lysate overexpressing ER81 as analyzed by qPCR, **e** ChIP analysis showing increased occupancy of ZIC3 on EBS on *Th* promoter in MB tissue lysate overexpressing ER81 as analyzed by qPCR. Mean ± SE of three independent biological triplicates (*n* = 3), ^*^p < 0.05, ^**^p < 0.01, ^***^p < 0.001. All the experiments [except (**a**)] were performed in embryonic mouse brain-derived cultures in vitro.

A comparative analysis of ZIC3 occupancy on target promoters showed that its binding to *Pitx3* was restricted to MB and not OB tissue (Fig. 8a-i). While ZIC3 preferentially binds to *Th* EBS in OB, it fails to do so in MB, and its choice of *Pitx3* promoter binding is exclusive to MB (Fig. 8a-ii, iii). Forced over-expression of *Er81* in MB derived neurons, which lacked ER81, could not cause any appreciable change in the expression of *Th* (Fig. 8b-i, ii). Interestingly, *Pitx3* was downregulated in response to the ectopic expression of ER81 (Fig. 8c-i, ii). To determine if this could be attributed to loss in binding of ZIC3 on *Pitx3* promoter, we performed ChIP in ER81 overexpressing condition and found decreased binding of ZIC3 to *Pitx3* promoter (Fig. 8d) with a collateral establishment of ZIC3 association with *Th* promoter similar to the scenario observed in OB neurons (Fig. 8e). All these results advocate a switch in molecular partners and targets of ZIC3 to ensure proper *Th* regulation in spatially distinct regions of brain.

## Discussion

Transcriptional codes directing MB [31] and forebrain TH-positive cells are categorical [32]. More often, the neurochemical and morphological properties of these neurons are governed by the spatial source of stem cells from which they originate [33]. OB interneurons are mainly contributed from the lateral ganglionic eminence (LGE) [34] and septum [22], from embryonic day 12 (E12) until birth, and in adulthood, they arrive from the subventricular zone (SVZ) [35]. While studies in the past have reported ER81 expression in OB TH-positive interneurons globally [36], ZIC1 and ZIC2 expressions were shown to be exclusive of TH-positive cells and restricted to CalR-positive GABAergic interneurons. Also, it was shown that ZIC1 and ZIC2 contribute to the generation of CalR-positive neurons by suppressing DAergic fate [37]. Recently, Qin et al. [22] showed using the ZIC3-LacZ model system that *Zic3-*expressing cells originate from the septum and migrate towards the OB to generate several cell types, including the DA interneurons [22]. In the present study, we demonstrate that ZIC3 regulates the expression of TH in OB by interacting with ER81, whereas in MB by binding to the promoter of PITX3. Studies in vivo in MB substantiated the in vitro observation of ZIC3 regulating the TH expression. To convincingly show that the regulation of *Th* by ZIC3 is a cell autonomous phenomenon, we performed spatial distribution studies, which suggested a sub-population of TH positive neurons to express ZIC3-accounting for about one third of the total DA neuron population, and this sub-set of neurons plausibly suggests the septal origin. Our results corroborate well with the previous report that showed retarded OB development in compound mutants of ZIC1 and ZIC3 [6]. DAergic interneurons of OB are distinct from those of other regional identities due to their partial GABAergic nature [38]. As the major source of these DAergic neurons of OB is adult neurogenesis, a role for ZIC3 in this event cannot be ruled out. As we did not notice any significant cell death (Data not shown) upon ZIC3 abrogation, the compromise in TH expression upon ZIC3 inhibition, is probably not the result of elimination of these neurons, but perhaps due to the acquisition of an alternative neurochemical identity, which is yet to be deciphered.

A few TFs in a spatially restricted manner regulate the neurogenesis of TH-positive cells [25, 39, 40]. While PITX3 is actively engaged in regulating TH expression in MB [41], its expression in OB is undetectable. NURR1, a TF essential for maintenance and differentiation of DA neurons [42, 43], though it is present in OB, lacks the ability to regulate the TH expression [44]. ER81 is expressed in the cortex during the embryonic day E15–P14 and is involved in the differentiation of the layer 5 cells of the cortex, however, no detectable expression is observed in the adult cortex [45]. ER81 is also not expressed in the ventral part of the MB in both embryonic and adult life [30]. While the present study showed ZIC3 to interact with ER81 and fine-tune the ER81-mediated TH expression in OB, the lack of ER81 and its incapability to influence the dopamine pathway [30] in MB compelled us to introspect the alternative mechanism of ZIC3-mediated TH regulation in MB. Our results bring to the fore, ZIC3 as a novel upstream regulator of PITX3. Having established ZIC3-mediated regulation of TH in OB and MB neurons, we set out to determine its preference for molecular partners. An evident displacement of ZIC3 from *Pitx3* promoter following its association with ectopically expressed ER81 in MB probably hints towards a higher affinity of ZIC3 to ER81.

None of the known TFs, such as NEUROD1, PAX6, PBX1, or ETS family member ER81, has been shown to differentially regulate the TH +ve neuron pool outside of OB. This novel bimodal control of TH expression probably contributes to rendering these neurons distinct from one another. It would also be intriguing to know how the role of ZIC3 can be extrapolated to several other pockets of TH-positive neurons throughout the brain. Studying these events could be clinically relevant as OB and MB DA neurons respond differently to neurodegenerative insults like those in PD. As hyposmia is one of the earliest symptoms in PD patients [46], any contribution of ZIC3 to olfactory perception could be instrumental in ascertaining its importance in DA neuron function and disease predisposition. The information obtained from this study may facilitate engineering pluripotent stem cell-derived and transdifferentiation-based approaches to derive TH+ve neurons.

## Materials and methods

### Animals

All animal experiments were performed post approval by the Animal Ethics Committee of Manipal Academy of Higher Education (MAHE) and Indian Institute of Science (IISc), Bangalore (IAEC/KMC/110/2022 and CAF/Ethics/731/2020). All animals were handled in compliance with the guidelines of the Association for the Assessment and Accreditation of Laboratory Animal Care.

### Generation of Zic3 null mice

Mice were housed in the AAALAC-accredited Indiana University School of Medicine Animal Facility, and experiments were approved by the Institutional Animal Care and Use Committee. *Zic3* null pups were generated by crossing *Zic3* heterozygous females with *Zic3* hemizygous males. Mice are maintained on a C57BL6 x 129 background. P0 pups were euthanized by CO_2_ inhalation followed by decapitation. The heads were fixed with 4% (*v*/*v*) paraformaldehyde in phosphate-buffered saline (PBS) overnight at 4 °C.

A portion of the tail was removed and lysed using the Hotshot lysis buffer for 10 min at 95 °C. The resulting DNA was genotyped using the *Zic3* genotyping assay previously described (Purandare, 2002).

### Cell line culture

HEK293T cells were grown in DMEM High glucose medium supplemented with 10% Fetal Bovine Serum (FBS), 1× non-essential amino acids (NEAA), and 1× PenStrep. The cells were cultured till 80% confluence, split in the ratio of 1:5. R1 mouse embryonic stem cells (mESCs)—a kind gift from Prof. Catherine Verfaillie, KU Leuven, were cultured on a layer of inactivated mouse embryonic fibroblasts (MEFs) in medium containing Dublecco’s Modified Eagle’s Medium (DMEM) high glucose-83.3%, FBS-16%, l-glutamine 2 mM, anti-anti-1%, NEAA-1×, sodium pyruvate-1%, β Mercaptoethanol (β-ME)-1.2% and supplemented with 8 ng/mL of leukemia inhibitory factor (LIF). Prior to differentiation, mESCs were plated onto the gelatin-coated surface and grown for 2 days in medium containing LIF.

### OB-neuron differentiation

mESCs were plated onto ultra-low attachment dishes in ESC medium devoid of LIF to generate embryoid bodies (EBs) for 4 days. Later, the EBs were gently dissociated and plated at a density of 1 million cells/well of a 6-well plate with gelatin coating and allowed to attach in LIF-deprived medium. Neural patterning was induced by culturing in N2 medium supplemented with 2.5 ng/mL fibronectin and Insulin-Transferrin-Selenite (ITS). Medium change was provided every alternate day. Post 6th day, the cells were grown in medium containing N2 supplement, basic Fibroblast Growth Factor (bFGF), Fibroblast Growth Factor 8 (FGF-8), and Purmorphamine for 4 days. Finally, the cytokines were withdrawn, and the cells were allowed to achieve terminal maturation for 6 days in basal N2 medium.

### Olfactory training in mice

To determine ZIC3 expression pattern in mice with olfactory enrichment, 3–4-week-old Swiss Albino female mice were selected and categorized into control and test groups. Control animals were housed in a completely odor-free environment and were prevented from exposure to any natural or artificial olfactory stimuli for 20 days. The test mice were directly exposed to various food-related olfactory stimuli for 1 h every alternate day, such that their environment was enriched with dopamine-stimulating odor molecules. Both groups were maintained on the same diet, and their bedding was changed every day. At the end of the olfactory training, animals were subjected to cervical dislocation, and OBs were harvested for either transcript or protein analysis.

### Generation of OB and MB neurospheres

E13-E15 mouse embryos were obtained from the Center for Cellular and Molecular Platforms (CCAMP), Bangalore, and were subjected to cervical dislocation. The embryos were dissected using a sterile scalpel blade to separate the OB and MB regions. The tissues were thoroughly washed with sterile 1× Phosphate Buffered Saline (PBS_ supplemented with 0.5× Pen-Strep. OB and MB tissues were minced into fine pieces separately and dissociated using 0.25% trypsin for 10 min at 37 °C. Trypsin was neutralized using 1:4 serum-containing medium, and the cells were spun at 200 g for 5 min at room temperature (RT). After discarding the supernatant, the pellet was resuspended in neurospheres medium comprised of 1:1 DMEM F12 with 1× N2 supplement and Neurobasal medium with 1× B27 supplement with 2% FBS, 100 mM Glutamax, 100 µM β-ME. The cells were plated onto ultra-low attachment plates for neurosphere formation. Medium change was performed every third day till day 12.

### Primary OB and MB neuron differentiation

To achieve DA neuron-like identity, OB or MB neurospheres were gently dissociated and plated onto 0.1% gelatin-coated dishes at a density of 10 × 10^5^ cells/well of a 6-well plate in neurospheres medium. The plated cells were cultured as neural progenitors for 4 days in vitro (4 DIV) and further differentiation was induced by providing medium containing 1:1 DMEM F12 and Neurobasal medium supplemented with 1× N2, 100 mM Glutamax, 100 µM β-ME, 20 ng/mL bFGF, 100 ng/mL FGF-8 and 3 µM Purmorphamine. Differentiation was continued for 4 DIV (8 DIV) with alternate day medium change. Cells were fixed at 4 DIV and 8 DIV and characterized for the presence of appropriate markers to assess the efficiency of differentiation. All cells were cultured in a humidified incubator (Thermo Fisher, USA) at 37 °C and 5% CO2.

### Transduction

For the delivery of shRNA constructs, we utilized lentiviral packaging. HEK 293Ts were transfected using lentiviral packaging vectors Ps Pax2 and PMD 2 G and the plasmid of interest. pLKO puro vector was used as the scrambled control for *Zic3* shRNA. The medium containing the lentiviral particles was collected at 48 h and 72 h post-transfection. The viruses were concentrated by incubating with 50% poly-ethylene glycol (PEG) 6000 solution for 24 h at 4° C on a rocker. The medium was spun at 250 g for 1 h at 4° C, and the pelleted viral particles were used for transducing the recipient cells.

### Transcript analysis: RNA isolation, cDNA synthesis, and PCR

RNA was isolated from cultured cells by the phenol-chloroform method using Trizol. The cells were washed with 1× PBS after decanting the medium and resuspended in Trizol. The RNA was isolated and stored at −80 °C until use. The isolated RNA was used to synthesize complementary DNA (cDNA) using a commercially available first-strand cDNA synthesis kit (# PGK162-A). The obtained cDNA was diluted 1:5 times using nuclease-free water and further used for polymerase chain reaction.

Conventional PCR was performed by using Takara Emerald PCR Master Mix and the amplicons were observed on a 1.7% agarose gel. Real time PCR was performed using SYBR Green mix (#RR820B) with low ROX as the reference dye in 7500RT PCR system (Applied Biosystems, USA). Expression of the housekeeping gene *Gapdh* was set as control and the relative fold change for the gene expression was calculated.

### Microarray analysis

The concentration and purity of the RNA extracted were evaluated using the Nanodrop Spectrophotometer (Thermo Scientific; 2000). The integrity of the extracted RNA was analyzed on the Bioanalyzer (Agilent; 2100). The samples for Gene expression were labeled using the Agilent Quick-Amp labeling Kit (p/n5190-0424). The total RNA was reverse transcribed at 40 °C using oligo dT primer tagged to a T7 polymerase promoter and converted to double-stranded cDNA. Synthesized double-stranded cDNA was used as a template for complementary RNA (cRNA) generation. cRNA was generated by in vitro transcription, and the dye Cy3 CTP(Agilent) was incorporated during this step. Labeled cRNA samples were fragmented at 60 °C and hybridized onto Agilent Mouse Gene Expression Microarray 8X60K. Fragmentation of labeled cRNA and hybridization were done using the Gene Expression Hybridization kit (Agilent Technologies, In situ Hybridization kit, Part Number 5190-0424). Hybridization was carried out in Agilent’s Surehyb Chambers at 65 °C for 16 h. The hybridized slides were scanned using the Agilent Microarray Scanner (Agilent Technologies, Part Number G2600D). Significant genes upregulated with fold change ≥ 1 (logbase2) and downregulated with fold change ≤ −1 (logbase2) in the test samples with respect to the control sample were identified in the case of mESCs differentiated to neuronal lineage samples. Biological analysis was performed for the differentially expressed genes based on their functional category and pathways using the biological analysis tool DAVID (http://david.abcc.ncifcrf.gov/). The microarray data have been deposited in Gene Expression Omnibus (GEO) and are accessible through GEO series accession number GSE221773 (Comparative analysis of gene expression in control and *Zic3* knockdown mESCs differentiated to neurons).

### Cloning

For *Zic3*-GST cloning, the 1401 bp (*Zic3*) was amplified using mESC cDNA as a template, and the amplicon was gel eluted using a gel elution kit (Abzyme). The vector backbone AIRAP9-PSMD2-GST (Addgene no. 21799) was digested using *HinDIII* and *XhoI* to remove the PSMD2 Coding DNA Sequence (CDS), and the amplified *Zic3* gene was cloned using the Infusion kit (Clontech, USA) as per the manufacturer’s instructions. To obtain various truncated versions of ZIC3, regions of CDS without one or more zinc finger domains were amplified and cloned between *HinD*III and *Xho*I regions of the GST tag vector. To clone ER81 downstream of a 3× FLAG tag, the FLAG-FUS WT vector backbone (Addgene no. 44985) was digested with *KpNI* and *XhoI* enzymes, and the FUS CDS was replaced by ER81 CDS, which was amplified using mouse OB cDNA.

### Immunofluorescence

Cells were washed thrice with 1× PBS and were fixed using 4% para-formaldehyde (PFA) for 20 min at RT and later washed thrice with 1× PBS. Permeabilization was done using 0.5% Triton-X 100 prepared in PBS for 30 min, followed by blocking using 20% FBS in PBS for 20 min. All steps were carried out at RT. Appropriate primary antibodies diluted in PBS were added to cells and incubated overnight at 4 °C. Cells were washed thrice with 1× PBS to remove any non-specific binding. Fluorescent tagged secondary antibodies were added to the cells at a concentration of 1:800 (diluted in PBS) for 1 h 30 min at RT. The cells were then washed with PBS and were counterstained with 4′,6-diamidino-2-phenylindole (DAPI) (1:10000 dilution) for 1 min and again washed with PBS. Cells were observed and imaged using an Olympus 1 × 73 inverted microscope under 20× magnification. Images were tinted and further processed using ImageJ software.

### Fluorescence-activated cell sorting (FACS)

Trypsinized cells were washed with 1× PBS and later fixed using 0.5% PFA. The samples were stored at 4 °C till use. Permeabilization was performed with 1× BD Permwash solution in PBS for 30 min at RT on a rocking platform. The non-specific sites were blocked using 20% FBS in 1× BD Permwash for 20 min at RT. Primary antibody was added in 1× BD Permwash at a dilution of 1:400 and incubated at 4 °C on a rocker overnight. The cells were washed with 1× PBS thrice, and secondary antibody was added in 1× BD Permwash at a dilution of 1:800 and incubated at RT for 1 h in the dark. The antibodies used have been listed in Supplementary Table 2. Washes were performed with 1× PBS thrice, the cells were resuspended in PBS, and the events were acquired on LSR II flow cytometer using 488 laser line or on FACS ARIA Fusion using 561 nm laser.

### Magnetic activated cell sorting (MACS)

About 10 × 10^6^ OB cells were resuspended in 70 μL of buffer containing 0.5% Bovine Serum Albumin (BSA) in 1× PBS. Further, 10 μL of FcR blocking reagent was added to the cells and incubated for 10 min at 4 °C. The antibody microbeads were added at a volume of 20 μL per tube, mixed thoroughly, and incubated for 15 min at 4 °C. The cells were washed with 2 mL of buffer and centrifuged at 300 g for 10 min, and the pellet was resuspended in 500 μL buffer. Magnetic separation was performed by passing the samples through MS columns. The unlabeled cells were obtained in the flow-through, and the magnetically labeled cells were plunged into a fresh tube. The separated cell populations were further labeled with PSA-NCAM antibody, and the procedure was repeated. Finally, the MACS separated cells were co-stained for TH and ZIC3 and were analyzed on a flow cytometer with single color and negative controls.

### Western blot analysis

Cells were lysed using Radioimmunoprecipitation assay buffer (RIPA) after adding protease inhibitor cocktail (PIC), sodium orthovanadate, and phosphatase inhibitors, and were sonicated. The samples were spun at 1200 g for 20 min at 4 °C, and the supernatant was collected. The isolated proteins were stored at −80 °C until further use. Proteins were fractionated on a 12% SDS PAGE, and the resolved proteins were transferred onto an activated PVDF membrane for 2 h at 100 mA using a semi-dry transfer apparatus. The blots were blocked using 5% skimmed milk and later incubated with respective primary antibodies at 4 °C, overnight on a rocking platform. Three washes were performed using 1× Tris-buffered saline with Tween 20 (TBST) (pH 7.4), after which, HRP-tagged secondary antibodies were added at a final concentration of 1:2000 in skimmed milk and incubated at room temperature for 1 h. After three washes with 1× TBST, the signal was developed using WesternBright ECL substrate (#K120445-D20) and captured in a Li-COR chemiluminescence detector. Uncropped immunoblot gels are shown in the Supplementary File.

### Chromatin immunoprecipitation (ChIP)

To assess binding of ZIC3 to *Th* promoter, a ChIP assay was performed using Sigma Imprint ChIP kit (Sigma-CHIP1-24RXN) according to the manufacturer’s protocol. Briefly, about 4 μg of ZIC3 or ER81 antibody was allowed to bind to the assay wells, washed with antibody buffer, and incubated on a rocker with 120 rpm for about 90 min. Excess antibody was removed by performing 6 washes with IP wash buffer and a final wash with Tris-EDTA buffer. The samples were prepared by making a single cell suspension of the OB tissue derived from an adult Swiss albino mouse. The cross-linking was performed using 37% formaldehyde in serum-free medium. The nuclear components were obtained by resuspending the cells in nuclei preparation buffer. The obtained DNA was resuspended in a shearing buffer with PIC (1×) and incubated on ice for 10 min. The sample was briefly vortexed and sonicated using 7 pulses thrice with an interval of 10 min between the cycles. The sheared chromatin was spun at 10,000×*g* for 10 min at 4 °C, and the supernatant was used for ChIP further. The lysate was diluted 1:1 with the dilution buffer and incubated in the antibody-bound wells on a rocker at 120 rpm for 90 min. The wells were washed with IP wash buffer, and the DNA release buffer containing proteinase K was added. The samples were incubated at 65 °C for 15 min. Reverse cross-linking was carried out for the samples by adding the buffer and incubating for 90 min at 65 °C. The solutions were added onto a DNA binding column and purified to obtain chromatin and used for targeted real-time PCRs. A 5% input of the lysate was used as a control, and the fold enrichment was plotted.

### Co-immunoprecipitation

OBs dissected from adult Swiss albino mice were minced and trypsinized using 0.25% trypsin for 10 min at 37 °C. The cell pellet was resuspended in IP lysis buffer containing Tris (20 mM, pH 8.0), NaCl (137 mM), NP-40 (1%), EDTA (2 mM), and protease inhibitors and incubated on a rocker at 120 rpm for half an hour. The cell lysate was spun at 1200 g for 10 min at 4 °C, and the supernatant was collected. The supernatant was pre-cleared by incubating with protein-A sepharose bead slurry on a rocking platform for 4 h at 4 °C. The samples were spun at 4 °C at 200 g for 5 min and the supernatant was collected as the pre-cleared lysate. The Protein-A slurry was incubated with 1–4 μg of the appropriate antibody on a rocking platform at 4 °C overnight. Later, the pre-cleared lysate was added to this mixture and incubated at 4 °C overnight on a rocker. Immunoprecipitation was performed by eluting the complex of sample antibody from the beads by inducing pH change with 0.2 M glycine (pH 2.0) followed by neutralization with 1 M Tris (pH 7.0). The immunoprecipitated samples were then used for Western blot analysis.

### Immunohistochemistry

Tissue was fixed in 4% PFA at 4 °C on a rocker overnight. Cryo-blocks were prepared and stored at −80° C until further use. Cryosection was performed using a Leica cryostat to obtain 10–12 µm thick sections on SuperFrost Plus Microscope Slides (Fisher Scientific) and stored at −20 °C until staining. The sections were washed thrice with 1× PBS and permeabilized with 0.1% Triton X100 for 1 h at RT, and blocked using 2% normal Donkey serum for 1 h at RT, and the sections were incubated with 1:100 to 1:500 primary antibody overnight at 4 °C. Washes were performed with 1× PBS and exposed to secondary antibody diluted in blocking solution to a final concentration of 1:600 and incubated for 2 h at room temperature. The sections were washed thrice with 1× PBS and counter-stained with 1:10000 DAPI. The stained neurons were imaged under a 20× objective in a Nikon TE 2000 inverted epi-fluorescence microscope.

### Luciferase assay

Cells were grown to 80% confluence and transfected with 50 ng TK-Renilla and 950 ng of *Th*-luciferase plasmids. After 24 h of transfection, medium was changed, and the cells were allowed to grow for another one day. The cells were harvested with an appropriate volume of passive lysis buffer and stored at −80 °C until further use. Luciferase assay was performed according to manufacturer’s instruction using dual luciferase assay kit (Promega Corporation). Readings were obtained using Ensight multiplate reader.

### Stereotaxy

Custom-made lentivirus carrying shRNA targeting *Zic3* was injected into 7-month-old wild-type BL-6 mice. Briefly, the mice were placed in an anesthesia chamber for induction with 2–3% isoflurane (Sosrane, NEON, Mumbai, India). Post induction, the mouse was placed on the stereotaxic apparatus (KOPF, TUNJUNG, CA, USA) with continuous supply of isoflurane of 1–1.5% for maintenance until the viral injections were completed. The injections were made in the MB using 10 µl Hamilton syringe fitted with a 28-gauge needle (Hamilton Company, Nevada, USA) using the following stereotaxic coordinates in mm with reference to bregma: anteroposterior (AP) = 3.0, mediolateral (ML) = 1.2, and dorsoventral (DV) = 4.5, with a flat skull position (Paxinos and Franklin). The viral injections were made at a speed of 0.1 μL per min (1 μL, of 5 × 10^12^ viral particles per mL) followed by an additional 5 min for complete diffusion of the virus and 3 min for slow withdrawal of the needle. Post injections, the mouse was warmed under an infrared (IR) lamp (245 V, 150 W) until being awakened and returned to the cage. The animals were maintained for 10 days and transcardially perfused. The brains were isolated, fixed in 4% paraformaldehyde (PFA) overnight, cryoprotected in 30% sucrose, frozen, and sectioned at 30 µm in a cryostat (Leica, CM 1850).

### Statistical analysis

All the experiments (unless mentioned in the figure legend) were performed in biological triplicates with added technical replicates wherever applicable. The values were averaged, and standard errors were calculated. Student’s *t*-tests were performed to determine the statistical significance, and the graphs were prepared using GraphPad Prism 8.0. Significance was decided as follows: ^*^p ≤ 0.05, ^**^p ≤ 0.005, ^**^p ≤ 0.001.

## Supplementary information

Supplemental Material

Original Data

## Data Availability

All the data supporting the findings of this study are available from the corresponding author upon reasonable request.
