# Supplementary material for: Zic3 enables bimodal regulation of tyrosine hydroxylase expression in olfactory bulb and midbrain-derived neurons
Source: Cell Death Discov. 2025 Apr 11;11:165. doi: 10.1038/s41420-025-02448-2 (PMC11992298; doi:10.1038/s41420-025-02448-2)
Supplement: Supplementary file 1 — Supplemental Material [file 41420_2025_2448_MOESM1_ESM.pdf]

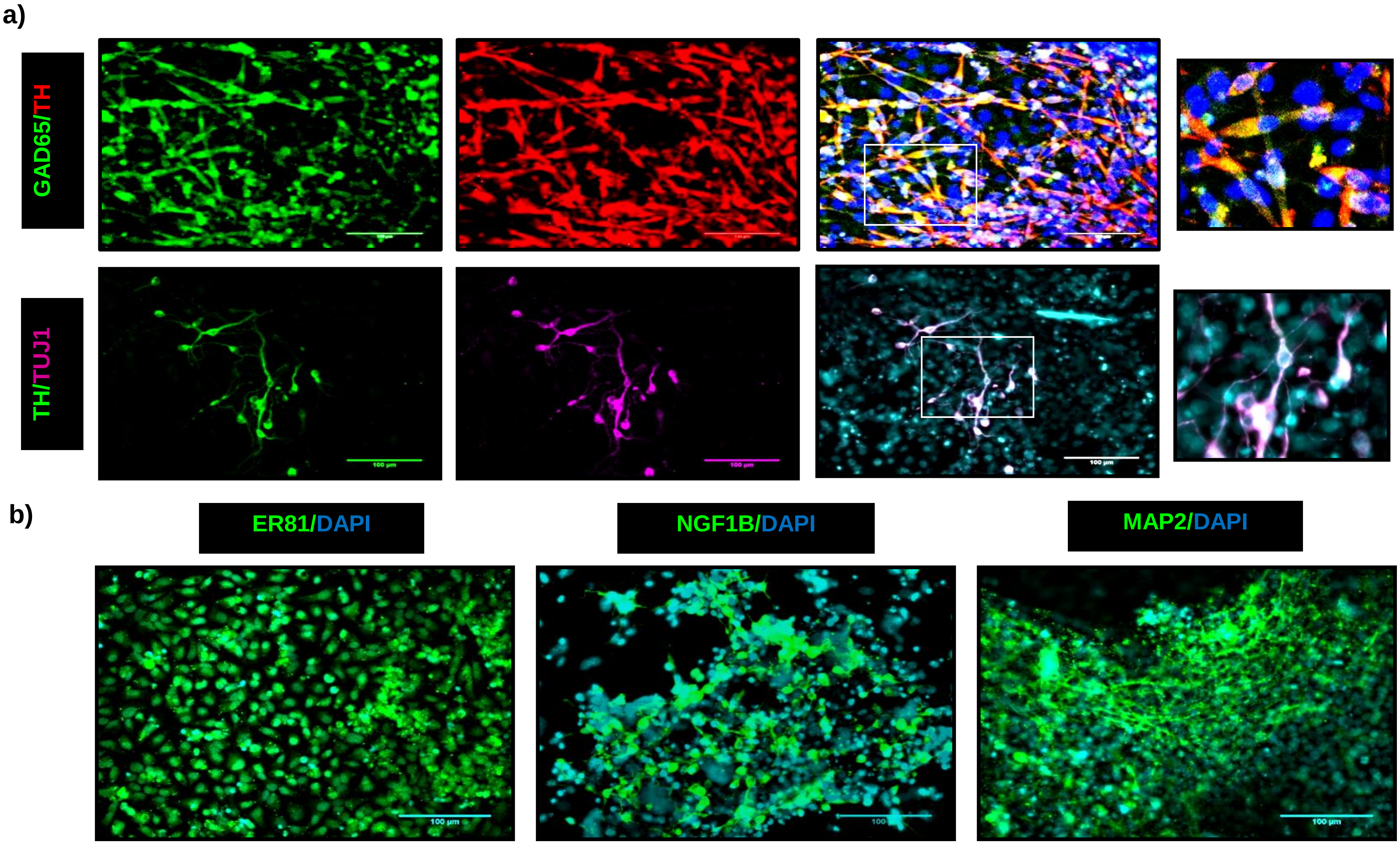
a)
GAD65/TH
TH/TUJ1
100 µm
b)
ER81/DAPI
NGF1B/DAPI
MAP2/DAPI
100 µm
100

Figure S1: mESCs efficiently differentiate to OB DA like neurons *in vitro*:
(a) Co-expression analysis of TH with GAD65 and TUJ1, b) staining for ER81, NGF1B and MAP2 in OB DA like neurons differentiated from mESCs. Scale bar represents 100 μm.

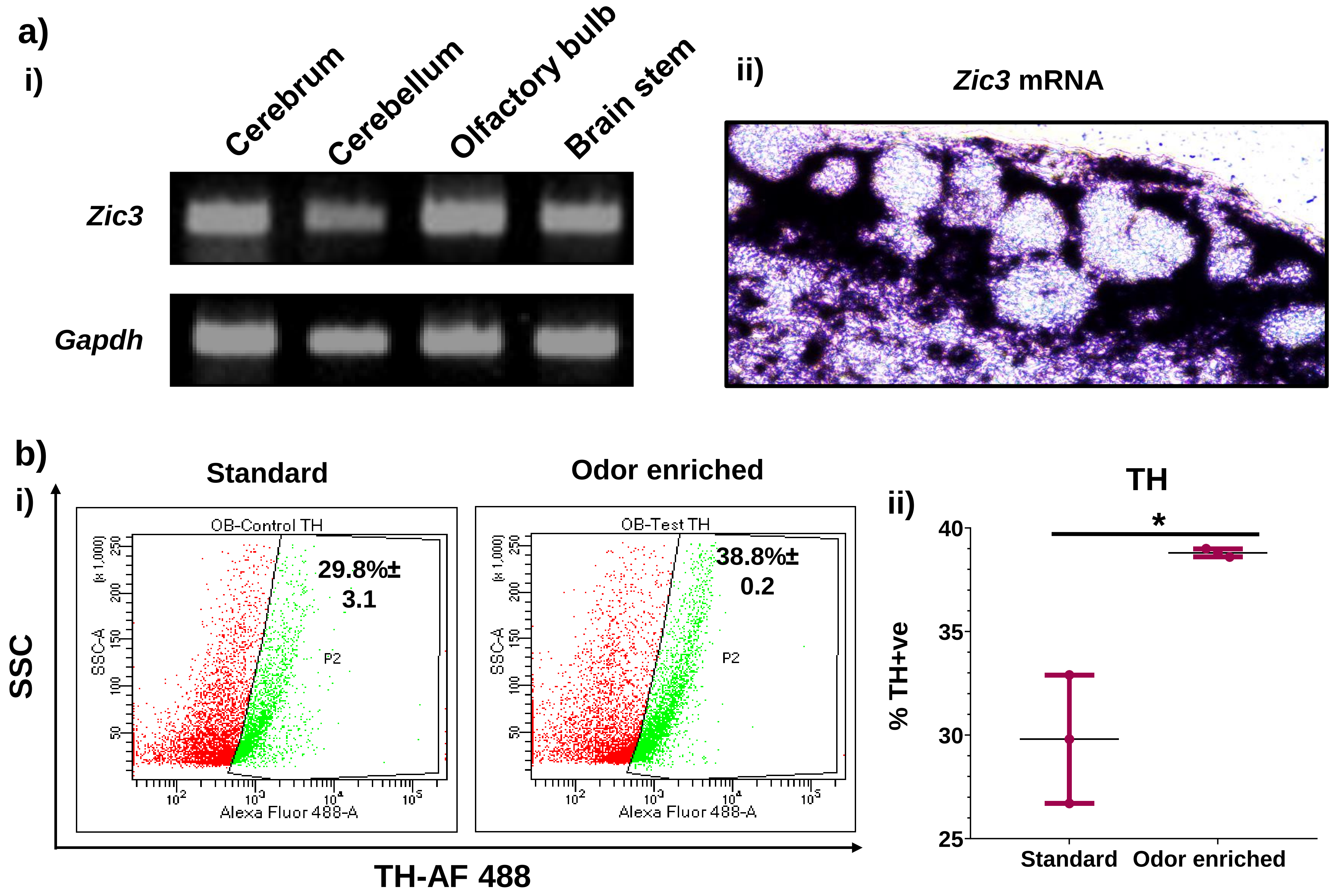

a)
i)
Cerebrum
Cerebellum
Olfactory bulb
Brain stem
Zic3
Gapdh
ii)
Zic3 mRNA
b)
i)
Standard
Odor enriched
SSC
OB-Control TH
29.8%±
3.1
P2
SSC-A
Alexa Fluor 488-A
OB-Test TH
38.8%±
0.2
P2
TH-AF 488
ii)
TH
*
% TH+ve
40
35
30
25
Standard
Odor enriched

Figure S2: ZIC3 is expressed in various regions of brain including olfactory bulb

(a) Transcript analysis of *Zic3* in various regions of brain (i), *In situ* hybridization showing Zic3 mRNA expression majorly in PGL of mouse OB (ii), (b) flow cytometry analysis (i) and quantification (ii) of ZIC3 in control and odor enriched mouse OB tissue. Mean+/-SE of three independent biological triplicates, * $p \leq 0.05$.

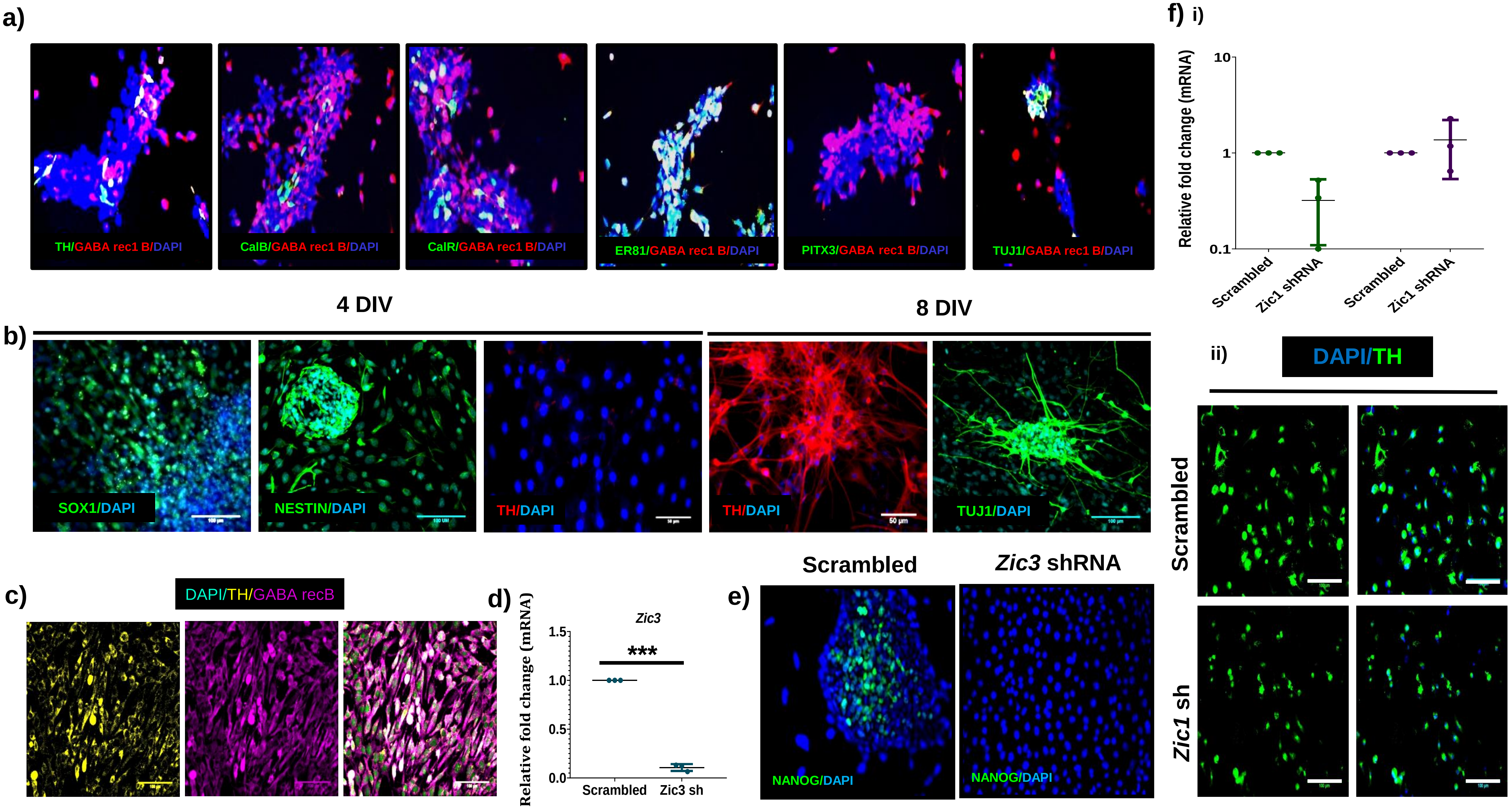

a)
TH/GABA rec1 B/DAPI
CalB/GABA rec1 B/DAPI
CalR/GABA rec1 B/DAPI
ER81/GABA rec1 B/DAPI
PITX3/GABA rec1 B/DAPI
TUJ1/GABA rec1 B/DAPI
4 DIV
8 DIV
b)
SOX1/DAPI
NESTIN/DAPI
TH/DAPI
TH/DAPI
50 µm
TUJ1/DAPI
c)
DAPI/TH/GABA recB
d)
Zic3
***
Relative fold change (mRNA)
1.5
1.0
0.5
0.0
Scrambled
Zic3 sh
e)
Scrambled
Zic3 shRNA
NANOG/DAPI
NANOG/DAPI
f) i)
Relative fold change (mRNA)
10
1
0.1
Scrambled
Zic1 shRNA
Scrambled
Zic1 shRNA
ii)
DAPI/TH
Scrambled
Zic1 sh

Figure S3: Characterization of Olfactory bulb primary neurons and analysis of effect of ZIC family member ZIC1 on OB differentiation

a) Immunofluorescence analysis of different OB genes along with demonstrating the absence of MB DA specific gene PITX3 which confirms the OB identity b) Expression analysis of progenitor genes SOX1 and NESTIN and matured gene TH at the end of 4 DIV and co-expression analysis of TH and TUJ1 at the end of 8 DIV, c) Co-expression analysis of GABA rec1B and TH, a hallmark of OB DA neurons at the end of 8 DIV. d) Transcript analysis of ZIC3 in OB primary neurons cultured in presence of either scrambled or ZIC3 shRNA construct, e) Expression of Nanog, the downstream target of ZIC3 in mESCs cultured in presence of either scrambled or Zic3 shRNA construct, f) Transcript analysis *Zic1* and *Th* and protein analysis of TH in primary OB neurons cultured in presence of either Scrambled or *Zic1* shRNA. Scale bar represents 100 µm. Mean+/-SE of biological triplicates, * $p \leq 0.05$, ** $p \leq 0.01$, *** $p \leq 0.001$

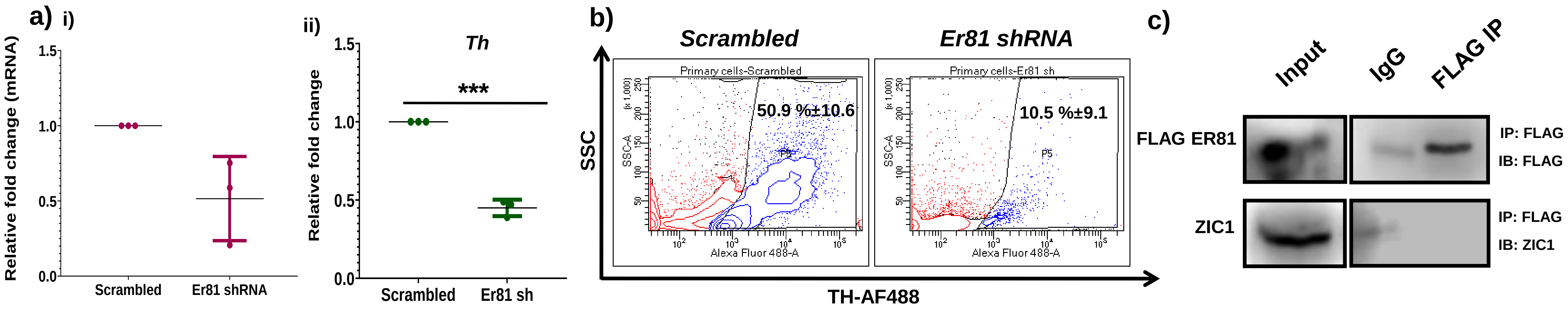
a) i)
Relative fold change (mRNA)
1.5
1.0
0.5
0.0
Scrambled
Er81 shRNA
ii)
Th
***
Relative fold change
1.5
1.0
0.5
0.0
Scrambled
Er81 sh
b)
Scrambled
Er81 shRNA
Primary cells-Scrambled
50.9 %±10.6
Primary cells-Er81 sh
10.5 %±9.1
SSC-A
Alexa Fluor 488-A
SSC
TH-AF488
c)
Input
IgG
FLAG IP
FLAG ER81
ZIC1
IP: FLAG
IB: FLAG
IP: FLAG
IB: ZIC1

Figure S4: ER81 regulates TH expression and fails to interact with ZIC1

a) Transcript analysis of *Er81* and *Th* and protein analysis of TH (b) in primary Ob neurons transduced with either scrambled or *Er81* shRNA. c) Co-immunoprecipitation analysis in HEK-293T cells overexpressing immunoreactive tags FLAG-ER81 and immunoprecipitation was performed using FLAG antibody and immunoblot performed using ZIC1 antibody. Mean+/-SE of biological triplicates, * $p \leq 0.05$, ** $p \leq 0.01$, *** $p \leq 0.001$

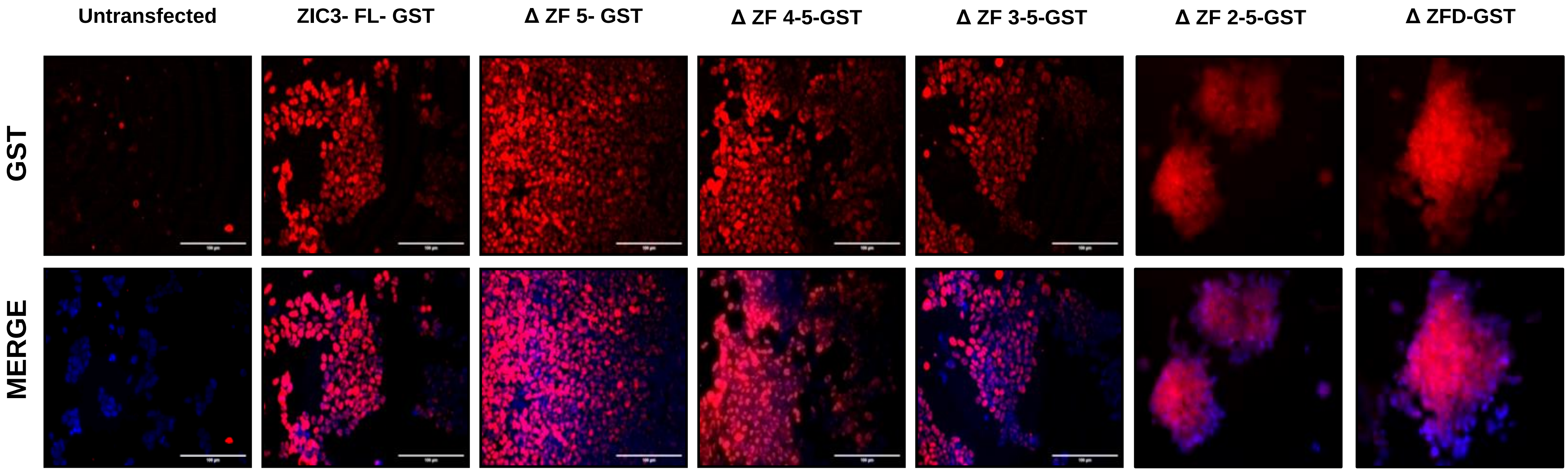

Figure S5: Zinc finger domain 1-2 is essential for nuclear localization of ZIC3

Immunofluorescence analysis of ZIC3 in cells overexpressing different deletion constructs of ZIC3 tagged to GST. Immunofluorescence was performed using GST antibody. Scale bar represents 100 µm

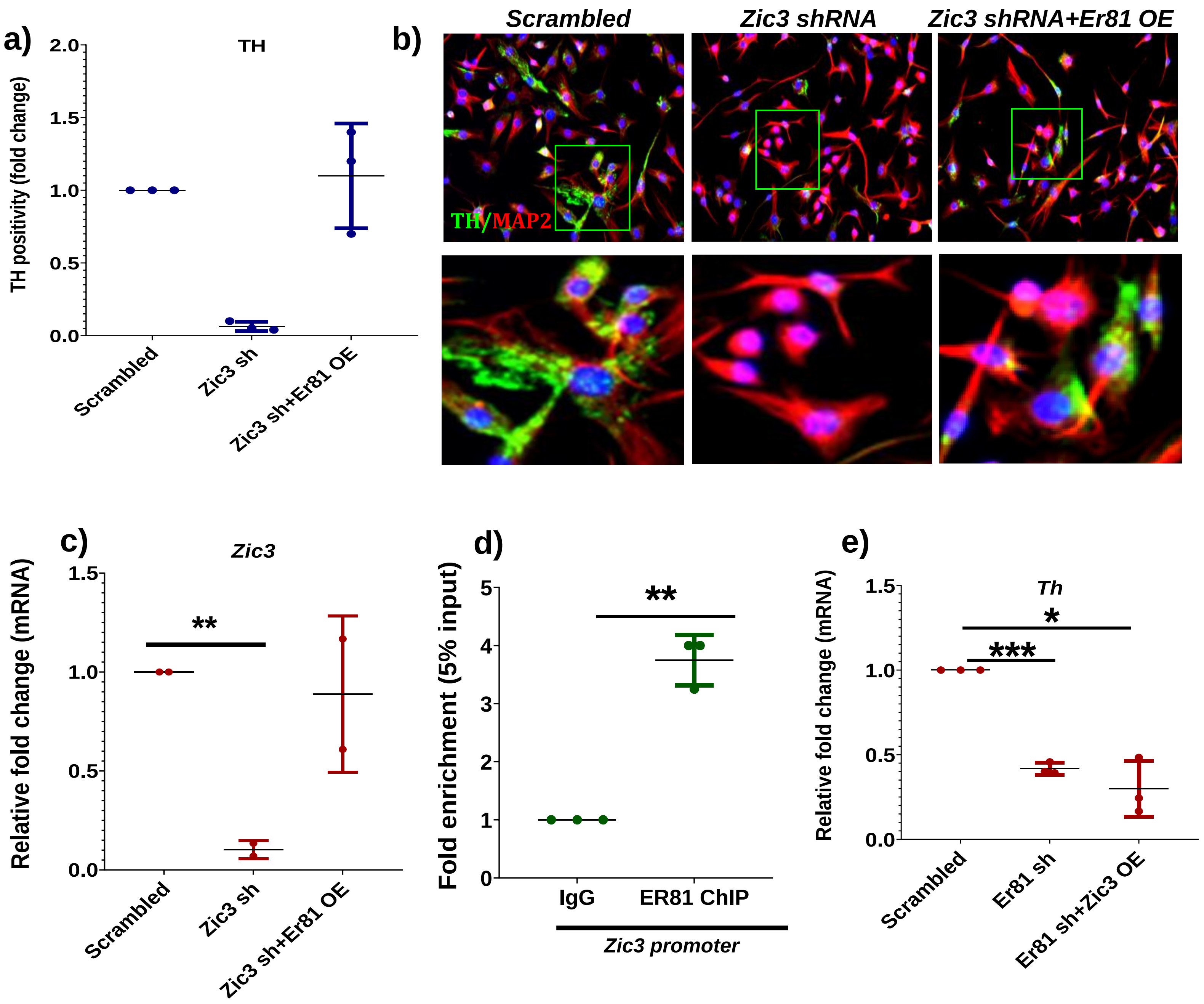

a)
TH
TH positivity (fold change)
2.0
1.5
1.0
0.5
0.0
Scrambled
Zic3 sh
Zic3 sh+Er81 OE
b)
Scrambled
Zic3 shRNA
Zic3 shRNA+Er81 OE
TH/MAP2
c)
Zic3
Relative fold change (mRNA)
1.5
1.0
0.5
0.0
**
Scrambled
Zic3 sh
Zic3 sh+Er81 OE
d)
Fold enrichment (5% input)
5
4
3
2
1
0
**
IgG
ER81 ChIP
Zic3 promoter
e)
Th
Relative fold change (mRNA)
1.5
1.0
0.5
0.0
*
***
Scrambled
Er81 sh
Er81 sh+Zic3 OE

Figure S6: ZIC3 fails to rescue *Th* expression in *Er81* knockdown condition

Effect of *Er81* over-expression on *Th* mRNA (a) and TH protein (b) levels in OB DA neurons with *Zic3* loss of function, (c) ChIP PCR with ER81 antibody showing binding of ER81 to *Zic3* promoter, (d) expression of *Th* in OB primary neurons with *Er81* shRNA and *Zic3* over-expression . Scale bar represents 100 µm. Mean+/-SE of biological triplicates, ** $p \leq 0.01$.

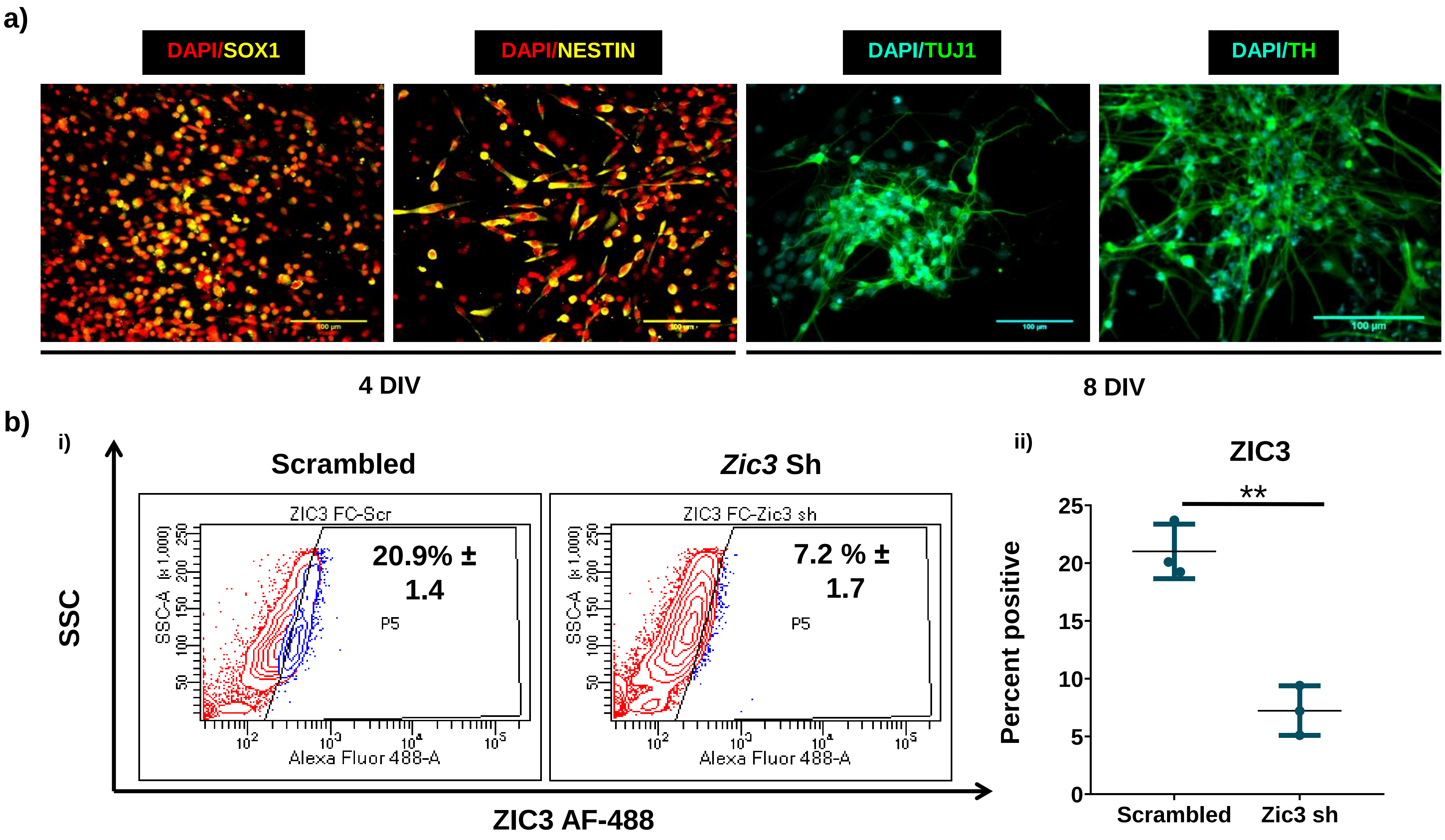

a)
DAPI/SOX1
DAPI/NESTIN
DAPI/TUJ1
DAPI/TH
4 DIV
8 DIV
b)
i)
Scrambled
Zic3 Sh
ZIC3 FC-Scr
ZIC3 FC-Zic3 sh
20.9% ± 1.4
7.2 % ± 1.7
P5
SSC-A (x 1,000)
Alexa Fluor 488-A
SSC
ZIC3 AF-488
ii)
ZIC3
**
Percent positive
Scrambled
Zic3 sh

Figure S7: Derivation of MB DA like neurons from primary neurospheres

(a) Immunofluorescence showing the expression of neural progenitor markers SOX1 and NESTIN in MB primary cells 4 DIV and matured neural markers TUJ1 and DA marker TH in 8 DIV, (b) knockdown efficiency of ZIC3 in MB DA neurons as shown flow cytometric staining (i) and quantification (ii). Scale bar represents 100 µm. Mean+/-SE of biological triplicates, ** $p \leq 0.01$.

Supplementary table 1

Primer name	Sequence	Amplicon size (bp)
mZic3	Forward primer:AAGATTTTTGCCCGCTCTG<br>Reverse primer:TATAGGGCTTGTCCGAGGTG	157
mGapdh	Forward primer: ACCACAGTCCATGCCATCAC<br>Reverse primer: TCCACCACCCTGTTGCTGTA	425
mOct 4	Forward primer: GAGGAGTCCAGGACATGAA<br>Reverse primer: AGATGGTGGTCTGGCTGAAC	153
mMap2	Forward primer: TCAGGAGACAGGGAGGAGAA<br>Reverse primer: GTGTGGAGGTGCCACTTTTT	112
mZic1	Forward primer: GCCCTTCAAAGCCAAATACA<br>Reverse primer: TTGCAAAGGTAGGGCTTGTC	252
mTh	Forward primer:AGGAGAGGGATGGAAATGCT<br>Reverse primer: GCGCACAAAGTACTCCAGGT	179
mGad65	Forward primer:AGATCGCCCCTGTATTTGTG<br>Reverse primer:GCATGGCATACATGTTGGAG	132
mEr81	Forward primer:TTCAGAACTCGGGTCTGCTT<br>Reverse primer:TGAGCTGTGTTTGGAGATGC	183
mNurr1	Forward primer:AGTCTGATCAGTGCCCTCGT<br>Reverse primer:GATCTCCATAGAGCCGGTCA	162
mEngrailed	Forward primer:GACTCTTCAGGCATCCAAGC<br>Reverse primer:GGGTCATCCAGTGCTGCTAT	258
mAadc	Forward primer:CTGATGTGGAGCCTGGCTAT<br>Reverse primer:GAGAAACCAATGCAGCCAAT	220
mVmat2	Forward primer:CCTCTGCTGGTGGTGCTATT<br>Reverse primer:CTTAATGGGGCAGTTGTGGT	164
mGch1	Forward primer:CAAGCAAGTCCTTGGTCTCA<br>Reverse primer:GAGGAACTCCTCCCGAGTCT	259
mNgf1b	Forward primer: TTCTGCTCAGGCCTGGTACT<br>Reverse primer: AATGCGATTCTGCAGCTCTT	204
mPitx3	Forward primer: GCAACTGGCCGCCCAAGG<br>Reverse primer: AGGCCCCACGTTCACCGA	84
m*Th* promoter luciferase	Forward primer: GAGGCCTCTTGGGATT<br>Reverse primer: CTGGTGGTCCCGAGTT	2559
m*Zic3* EBS	Forward primer: AAGCTGACAGGATCCAAAC<br>Reverse primer: TAAATCATGCAAATGAATTC	180
Th 4 (+7 to -99 bp)	Forward primer: TGGATGCAATTAGATCTAATGGGACG<br>Reverse primer: TGGGCATAGTGCAAGCTGGTGGTCCC	106
Th 3 (-49 to -245 bp)	Forward primer: GGATCTTTGTGTAAGTGG<br>Reverse primer: AGTTAAGAGTATCCTGAAC	196
Th 2 (-246 to -434 bp)	Forward primer: AAAGCAGAGGTCTGTCCC<br>Reverse primer: GTCCTATGAGACACAGAA	189
Th 1 (-435 to -629 bp)	Forward primer: CTGCCTGAGGACCCAGCC<br>Reverse primer: GTTCATGTTAGGAAGGC	195
m*Pitx3*ChIP	Forward primer: CCAAATCCTGCTTTCTCC<br>Reverse primer: CTATTCCAGTCCTCGTGC	185

m*Zic3* FL GST	Forward primer: ATGACGATGCTCCTGGAC<br>Reverse primer: GACGTACCATTCGTT	3366
m*Zic3* ΔZF5 GST	Forward primer: ATGACGATGCTCCTGGAC<br>Reverse primer: CGAGGTGTGCACATGCATG	2273
m*Zic3* Δ ZF4-5 GST	Forward primer: ATGACGATGCTCCTGGAC<br>Reverse primer: ACCTGTATGGGTCCTCTTG	1059
m*Zic3* ΔZF3-5 GST	Forward primer: ATGACGATGCTCCTGGAC<br>Reverse primer: GCCAGTGTGCACTCGGATA	969
m*Zic3* Δ ZF2-5 GST	Forward primer: ATGACGATGCTCCTGGAC<br>Reverse primer: GTTGTTCTGCTCCGGGCC	879
mHA *Zic3* ZFD	Forward primer: GAGCTGTCCTGTAAGTGG<br>Reverse primer: TCAGACGTACCATTCGTTA	2625
m*ER81* FLAG	Forward primer: ATGGATGGATTTTA<br>Reverse primer: TTAGTACACGTATC	1455
m*Zic3* promoter GFP	Forward primer: ACCAGGGGGAAGAGTGGTG<br>Reverse primer: GGGGAACCACGGGGCCAG	1451

Supplementary table 2

Antibodies	Source	Identifier
Goat anti ZIC3	Santa Cruz	#SC-28156
Rabbit anti ZIC3	ABclonal	#PA5-97216
Rabbit anti TH	ABclonal	#A12756
Mouse anti β-ACTIN	Santa Cruz	#SC-47778
Rabbit anti NANOG	Millipore	#AB9220
Chicken anti MAP2	Abcam	#ab5392
Rabbit anti TUJ1	Abcam	#ab18207
Mouse anti GAD65	Abcam	#ab26113
Rabbit anti ER81	Abzyme	#AB-PA002391
Rabbit anti NGF1B	CusaBio	#CSB-PA003493
Mouse anti NESTIN	BD Pharmingen	#556309
Mouse anti GABA B receptor 1	Abcam	#ab55051
Rabbit anti CALBINDIN	ABclonal	#A0802
Rabbit anti CALRETININ	ABclonal	#A9807
Rabbit anti PITX3	ABclonal	#A19261
Goat anti SP8	Santa Cruz	#SC-104661
Sheep anti PAX6	R & D biosystem	#AF8150
Mouse anti DDDDK-tag (Anti-FLAG)	ABclonal	#AE005
Rabbit anti HA-tag	ABclonal	#AE036
Rabbit anti GST	ABclonal	#AE006

Rabbit anti SOX1	ABcam	#ab109290
Anti PSA-NCAM	Miltenyi Biotec	#120-004-201
Anti A2B5	Miltenyi Biotec	#120-005-044
Chicken anti GFP	Aves labs	#GFP-1020
Rabbit anti TH	EMD Millipore	#AB152
Rabbit anti mouse FITC	Sigma Aldrich	#AP160F
Goat anti mouse AF594	ThermoFisher Scientific	#11005
Donkey anti rabbit AF594	ThermoFisher Scientific	#21207
Donkey anti rabbit AF488	ThermoFisher Scientific	#21206
Goat anti chicken AF488	Invitrogen	#A-11039
Rabbit anti mouse HRP	Southern Biotech	#6170-05
Goat anti rabbit HRP	Abcam	GR209629-6
Chicken anti goat FITC	Sigma Aldrich	#AP163F

Figure 1.e (i)

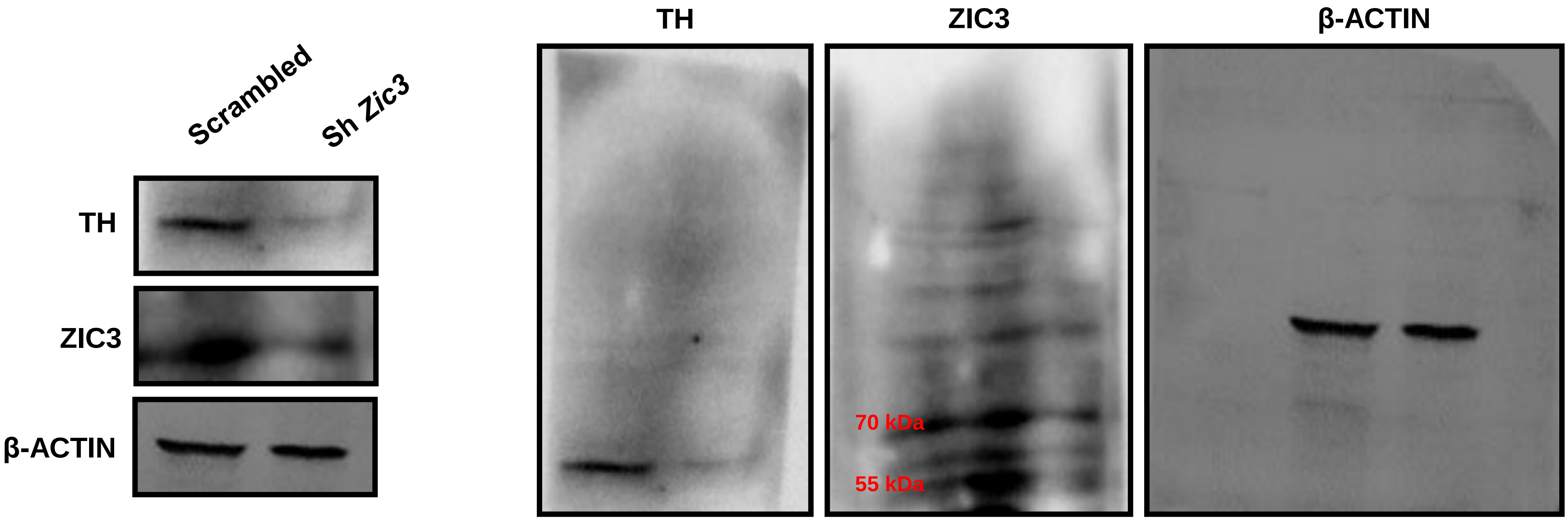

Figure 4.f

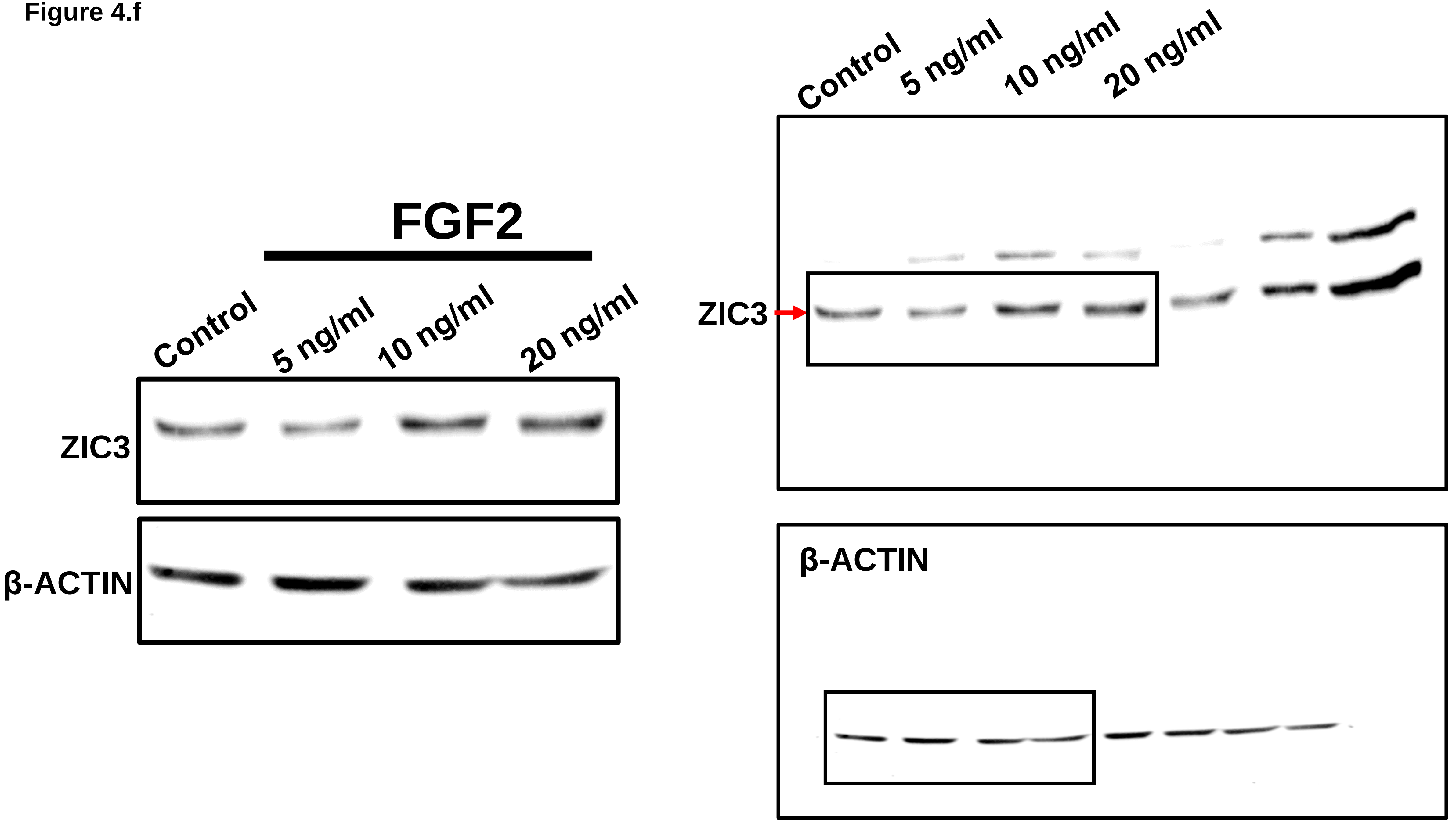

FGF2
Control
5 ng/ml
10 ng/ml
20 ng/ml
ZIC3
β-ACTIN
Control
5 ng/ml
10 ng/ml
20 ng/ml
ZIC3
β-ACTIN

Figure 4.f

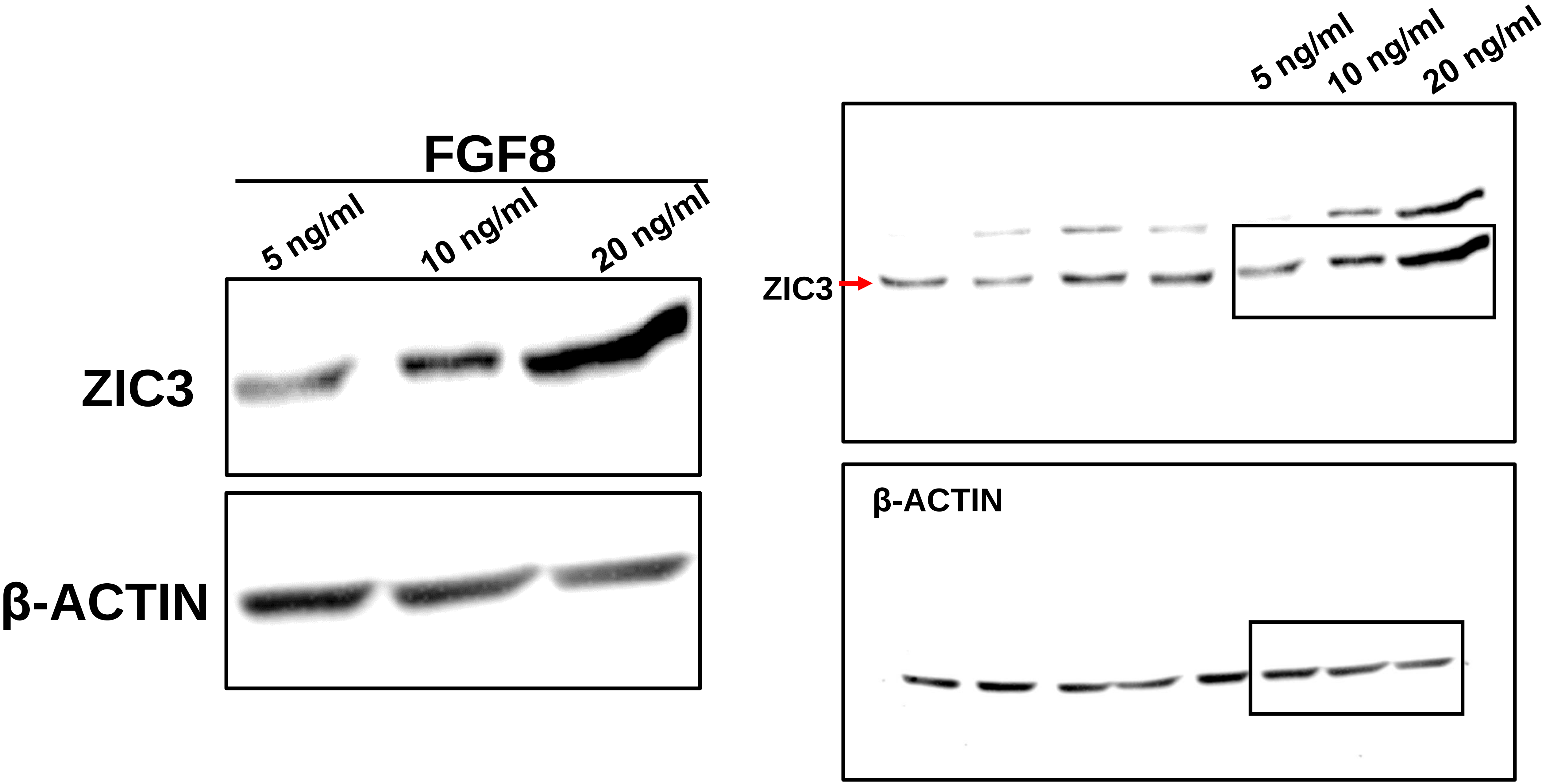

FGF8
5 ng/ml
10 ng/ml
20 ng/ml
ZIC3
β-ACTIN
5 ng/ml
10 ng/ml
20 ng/ml
ZIC3
β-ACTIN

Figure 4.f

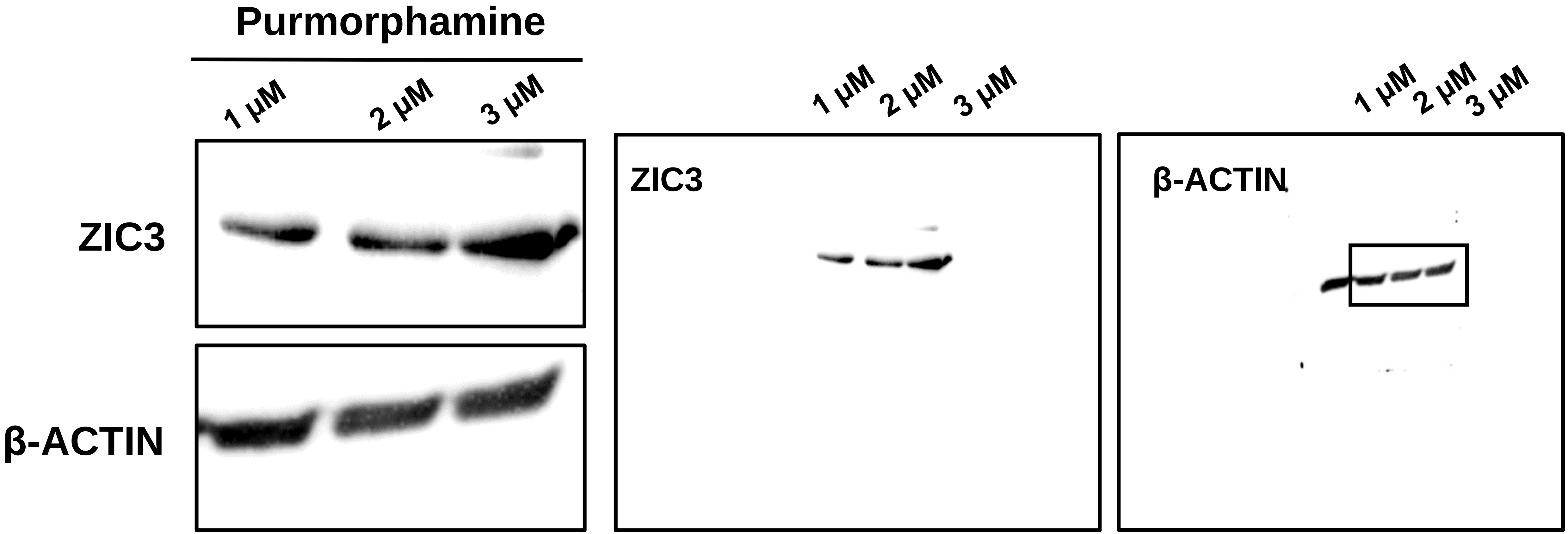

Purmorphamine
1 µM
2 µM
3 µM
ZIC3
β-ACTIN
1 µM
2 µM
3 µM
ZIC3
1 µM
2 µM
3 µM
β-ACTIN

Supplementary Figure S4.c

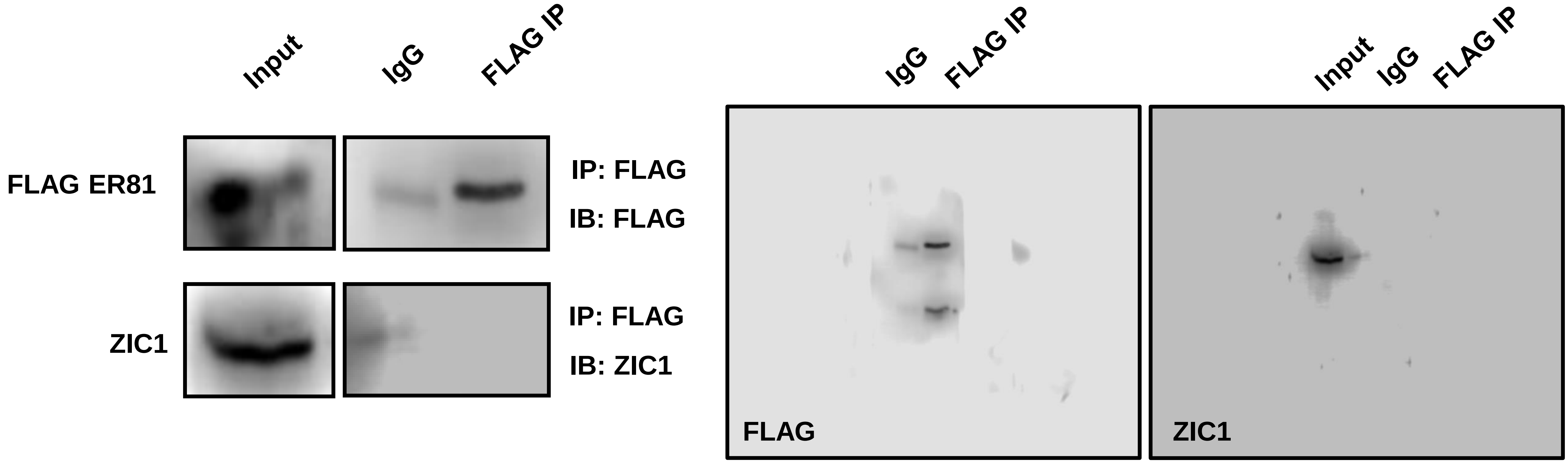

Figure 5.d (i)

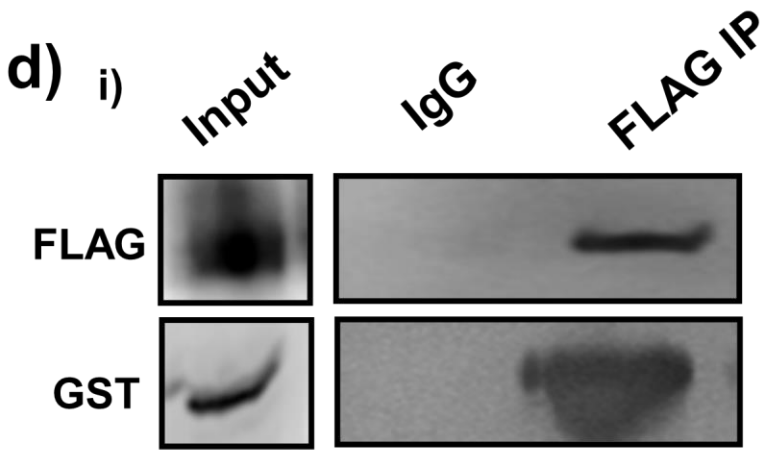

Figure 5.d (ii)

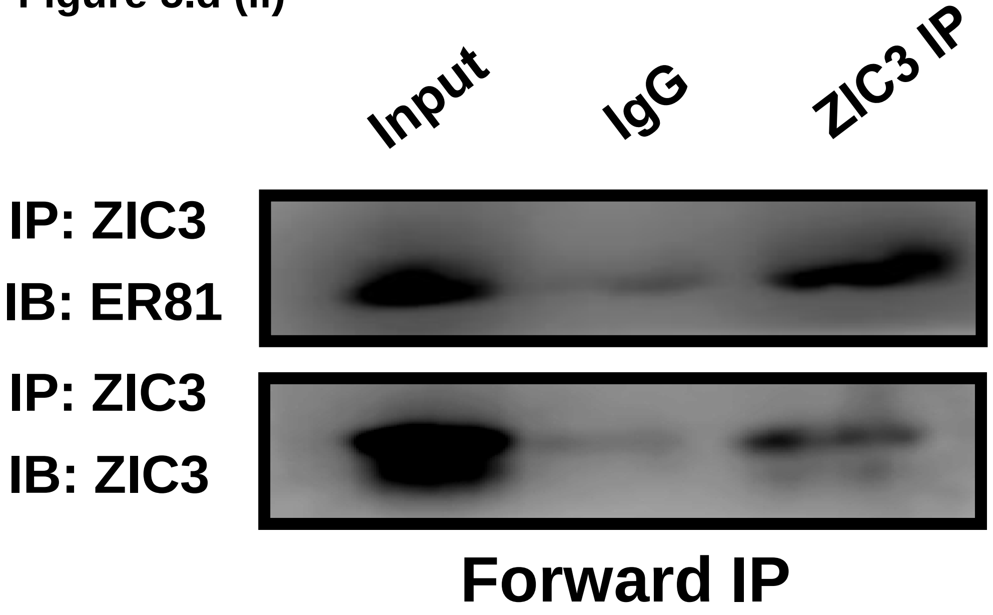

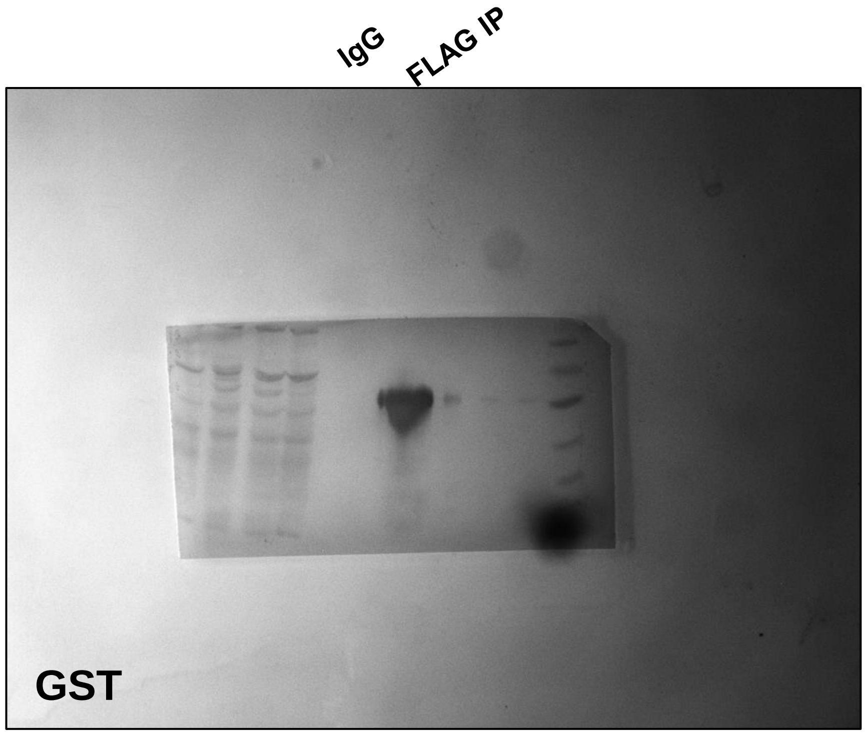

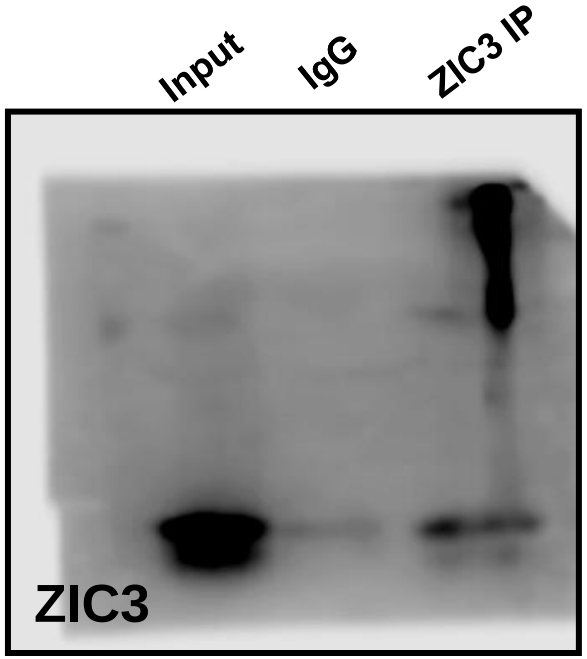

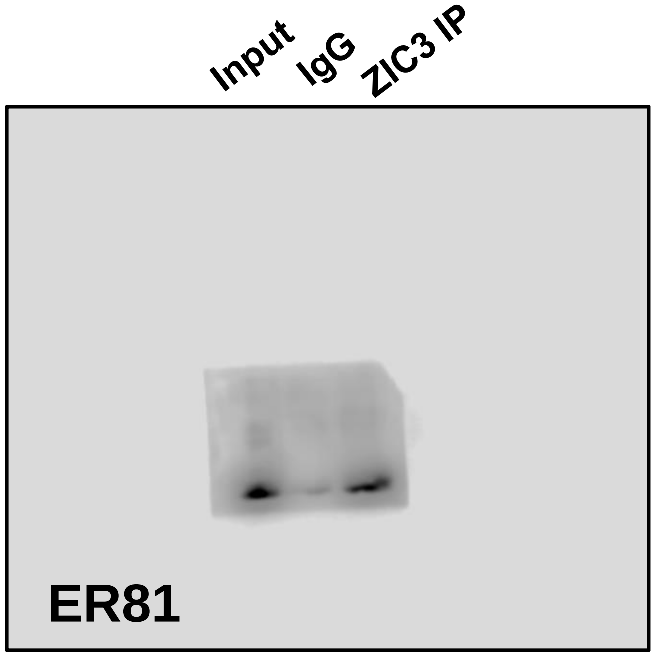

Figure 5.d (ii)

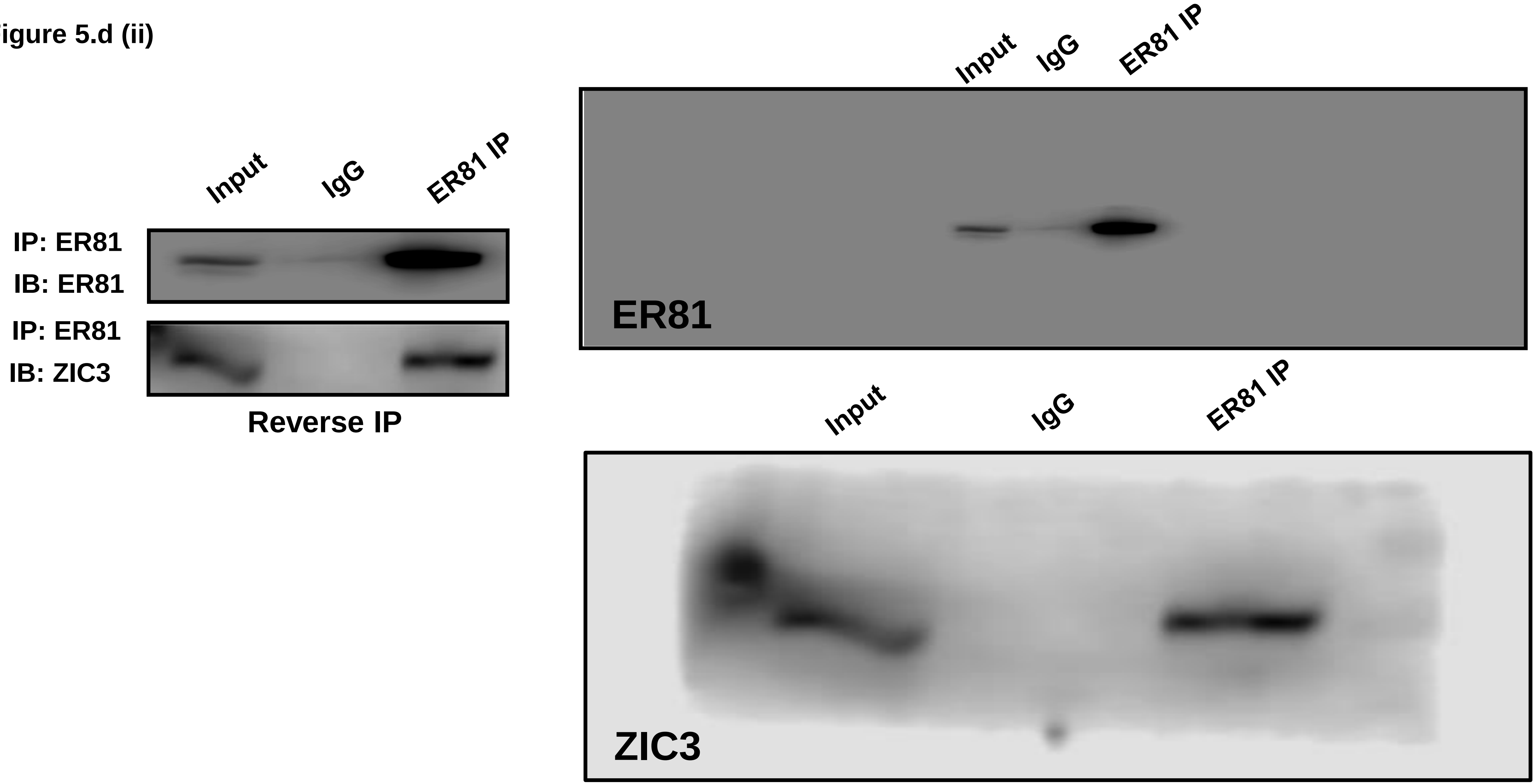

Input
IgG
ER81 IP
IP: ER81
IB: ER81
IP: ER81
IB: ZIC3
Reverse IP
Input
IgG
ER81 IP
ER81
Input
IgG
ER81 IP
ZIC3

Figure 5.e (i)

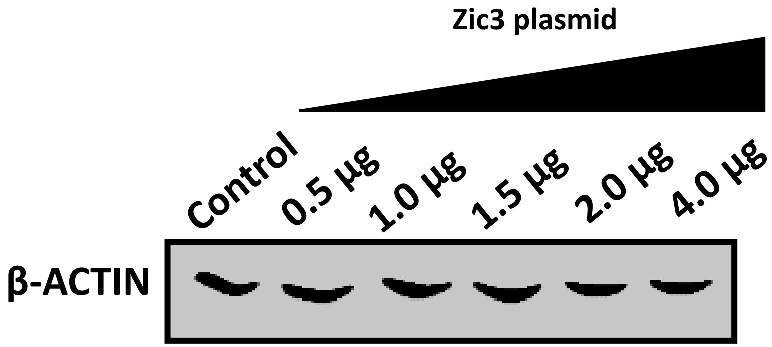

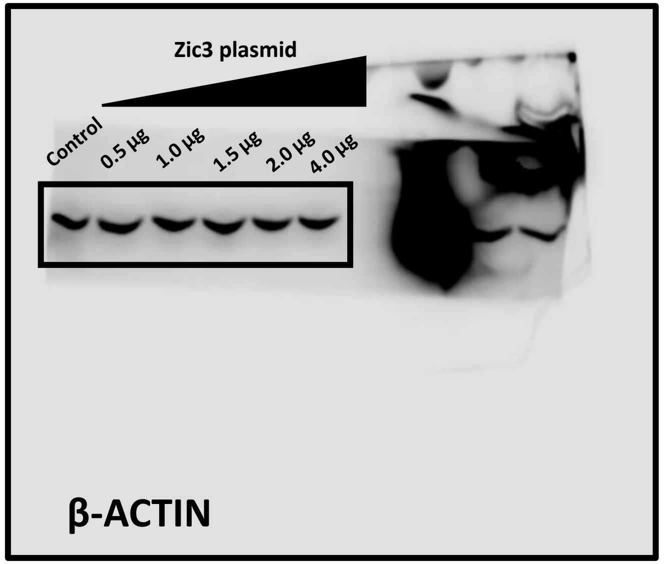

Figure 5.e (ii)

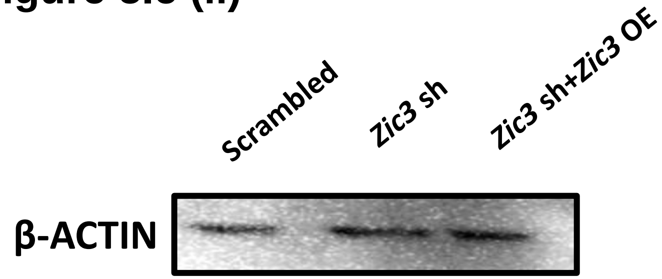

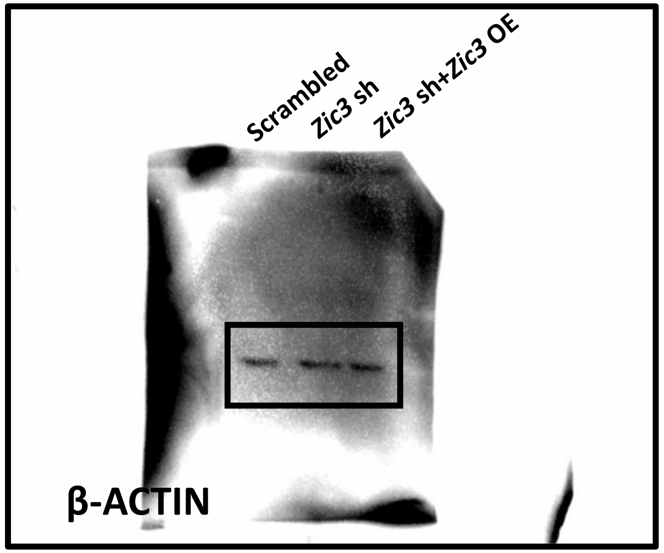

Figure 5.e (iii)

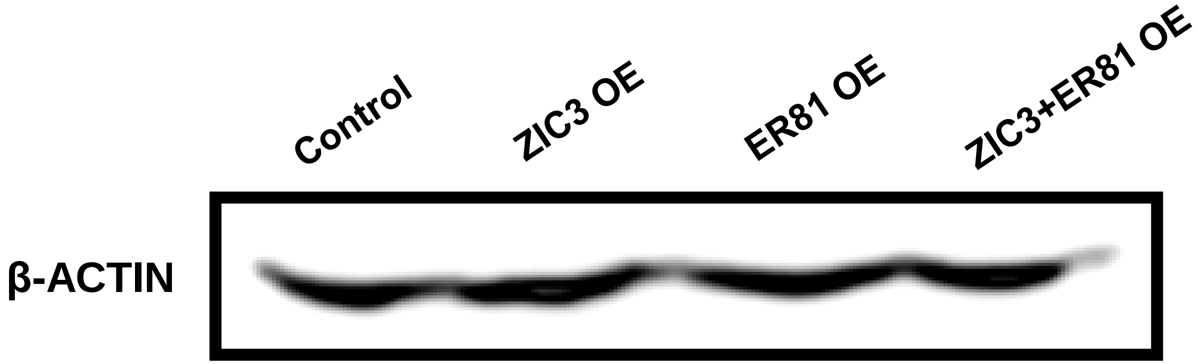

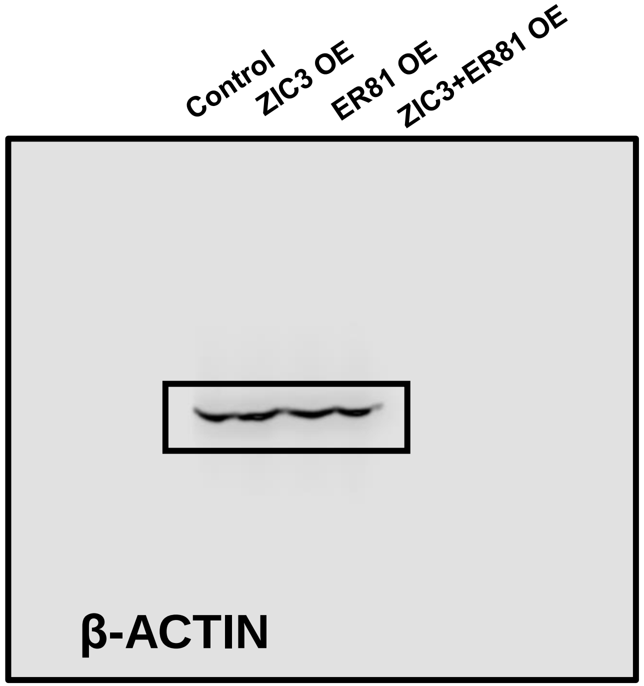

Figure 5.f (i)

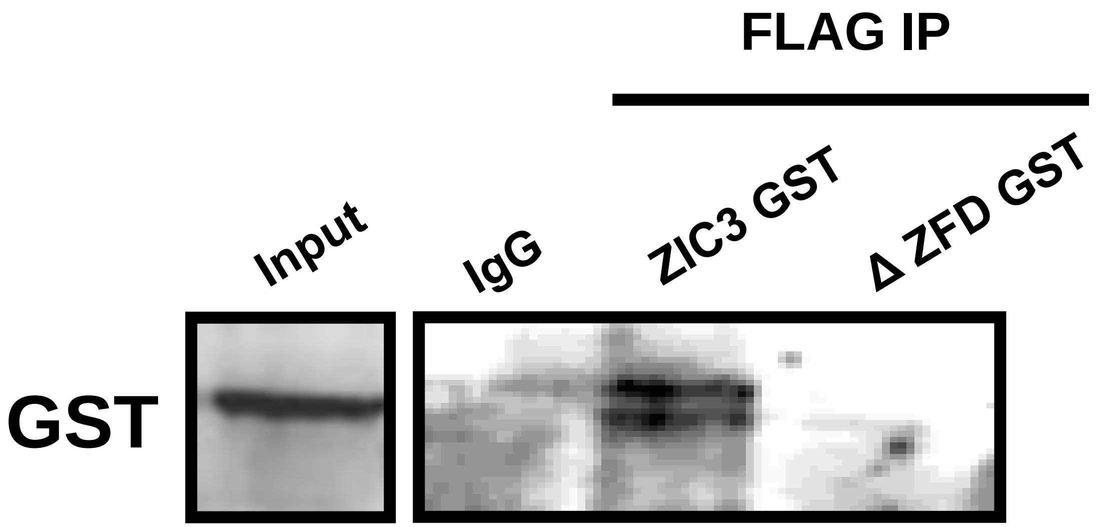
FLAG IP
Input
IgG
ZIC3 GST
Δ ZFD GST
GST

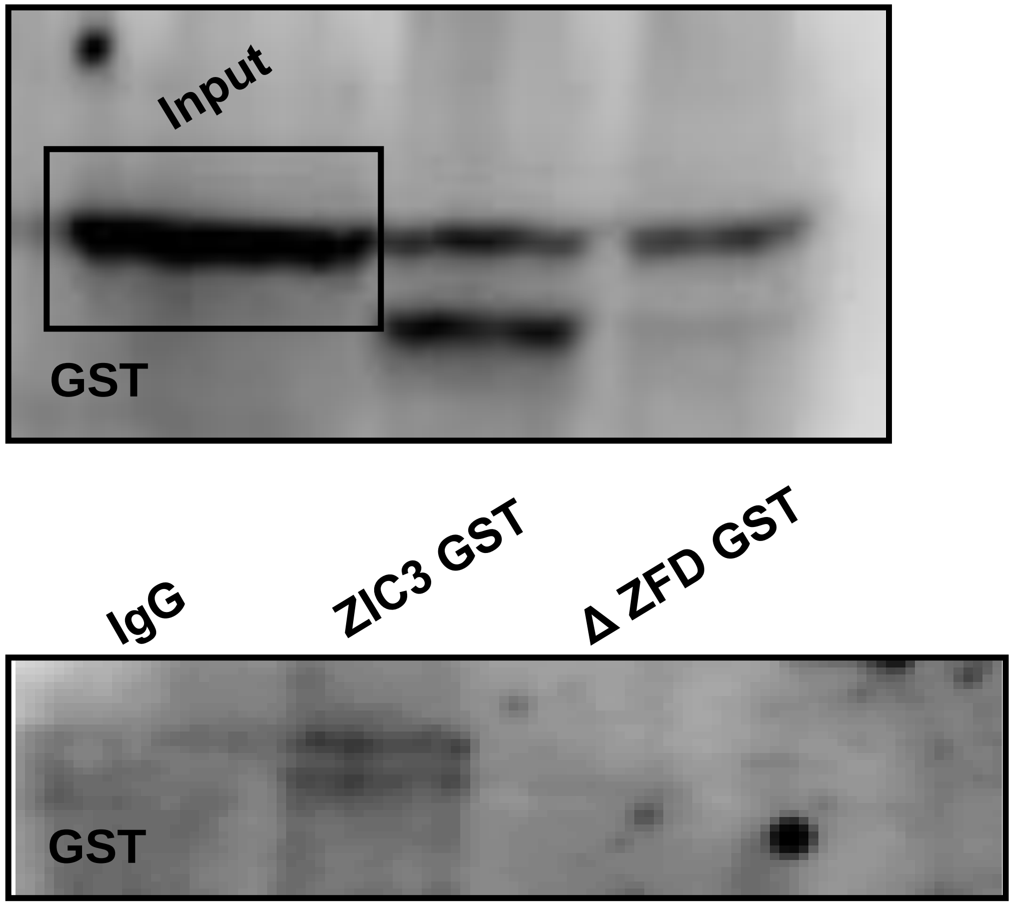
Input
GST
IgG
ZIC3 GST
Δ ZFD GST
GST

Figure 5g

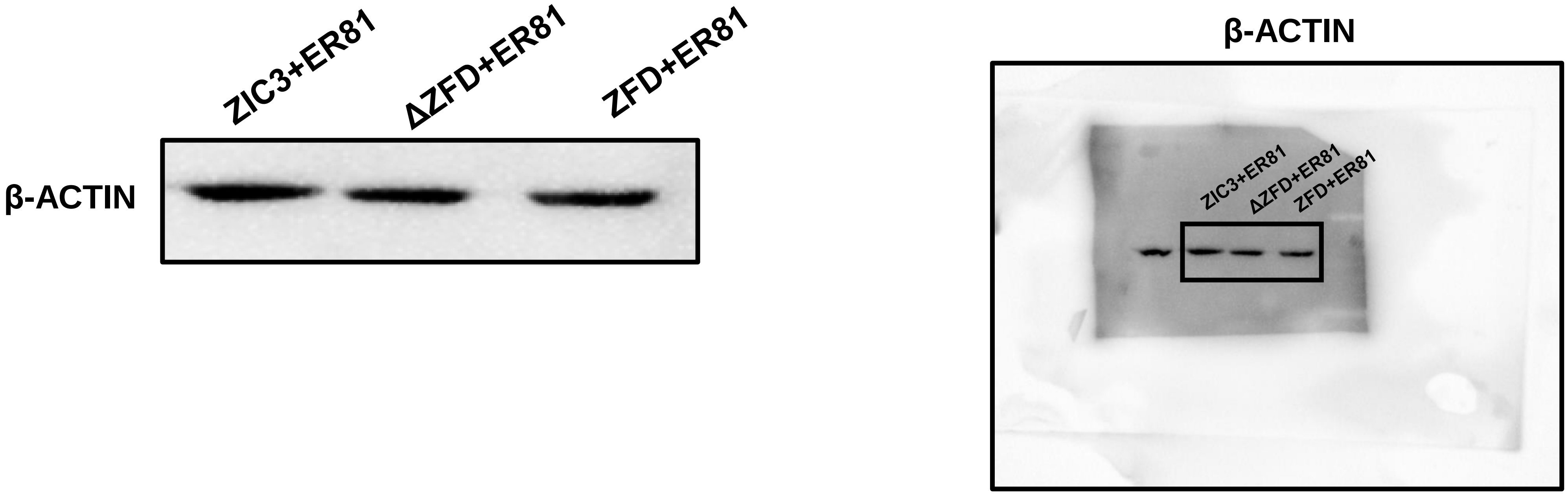
ZIC3+ER81
ΔZFD+ER81
ZFD+ER81
β-ACTIN
β-ACTIN
ZIC3+ER81
ΔZFD+ER81
ZFD+ER81
