# Supplementary material for: Zic3 enables bimodal regulation of tyrosine hydroxylase expression in olfactory bulb and midbrain-derived neurons
Source: Cell Death Discov. 2025 Apr 11;11:165. doi: 10.1038/s41420-025-02448-2 (PMC11992298; doi:10.1038/s41420-025-02448-2)
Supplement: Supplementary file 2 — Original Data [file 41420_2025_2448_MOESM2_ESM.pdf]

Figure 1.e (i)

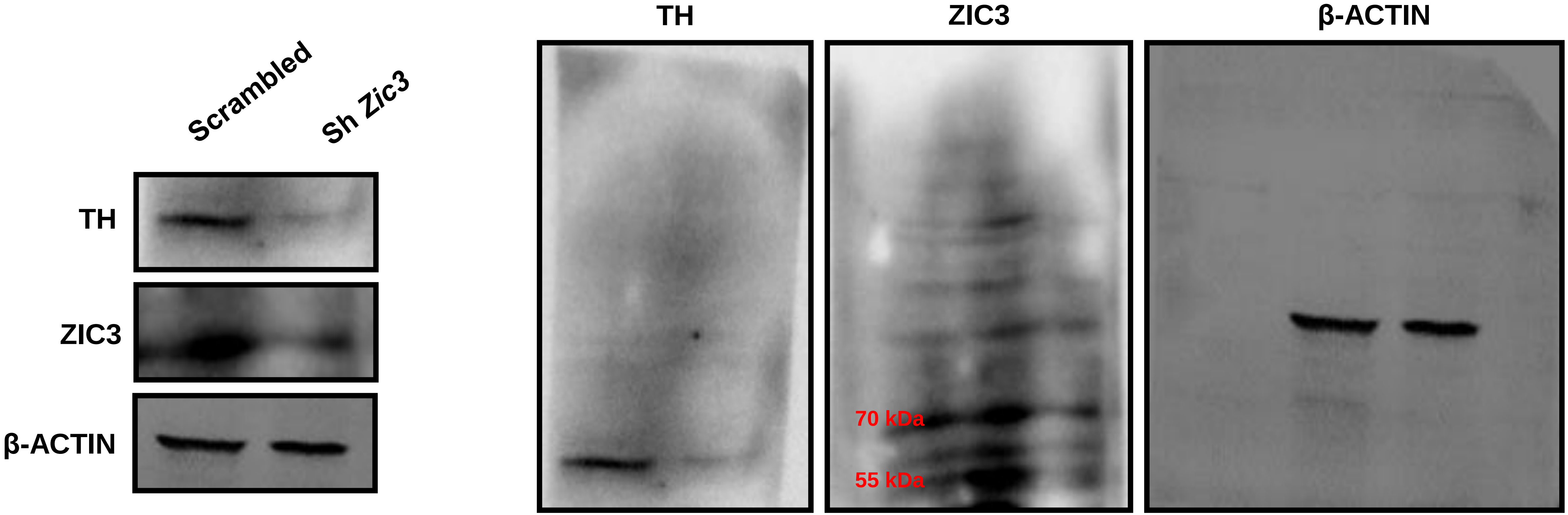

Figure 4.f

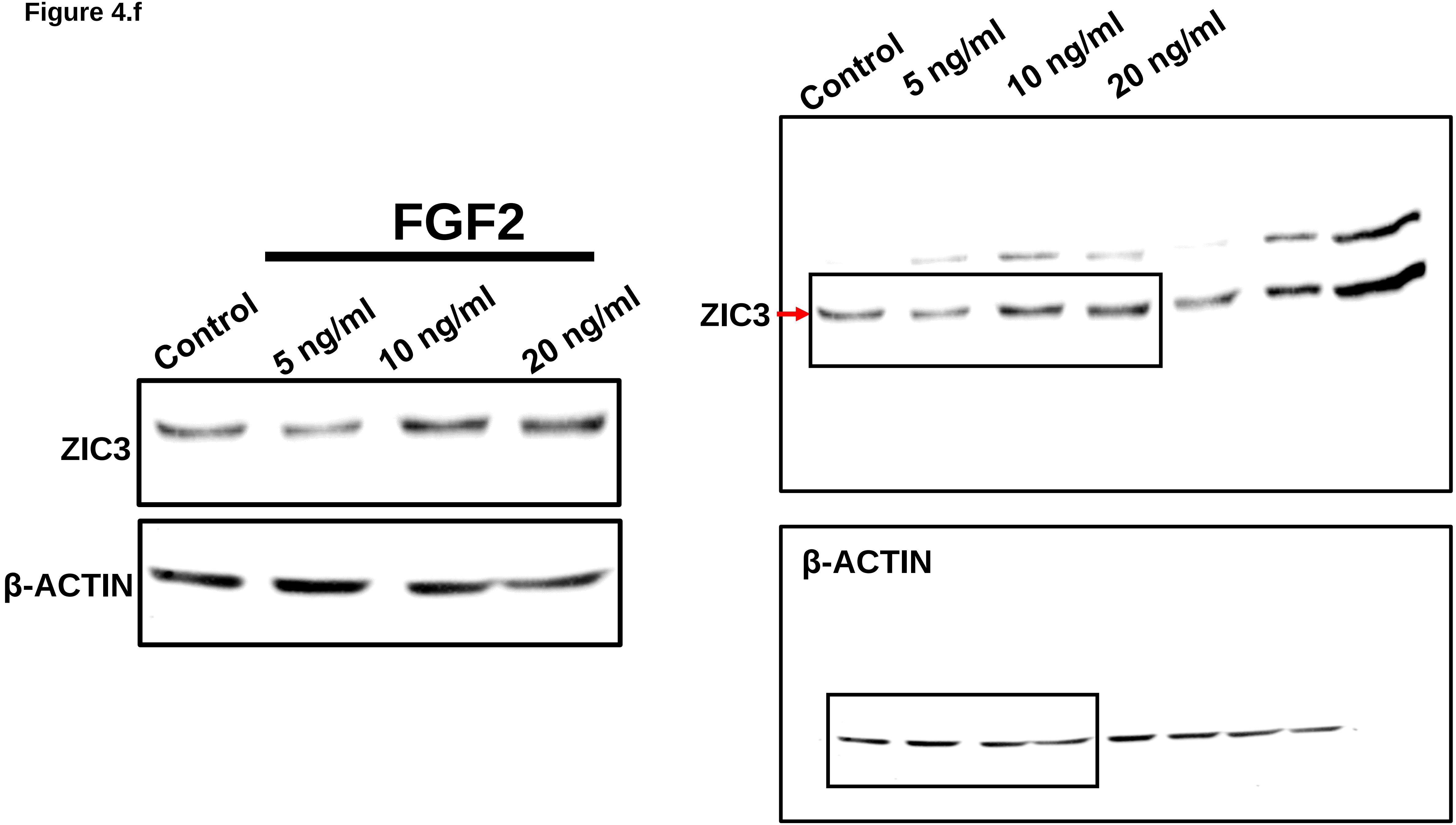

FGF2
Control
5 ng/ml
10 ng/ml
20 ng/ml
ZIC3
β-ACTIN
Control
5 ng/ml
10 ng/ml
20 ng/ml
ZIC3
β-ACTIN

Figure 4.f

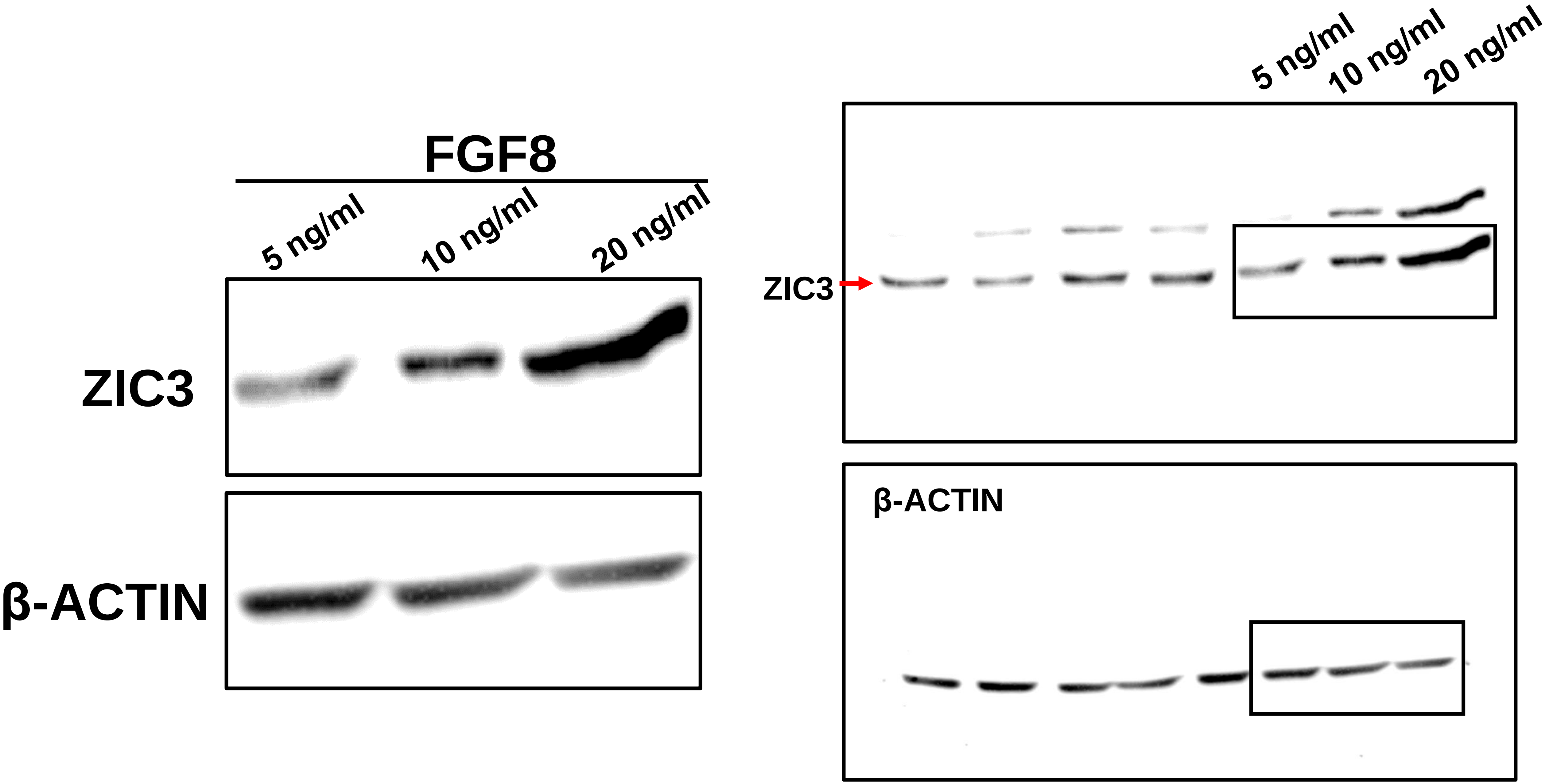

FGF8
5 ng/ml
10 ng/ml
20 ng/ml
ZIC3
β-ACTIN
5 ng/ml
10 ng/ml
20 ng/ml
ZIC3
β-ACTIN

Figure 4.f

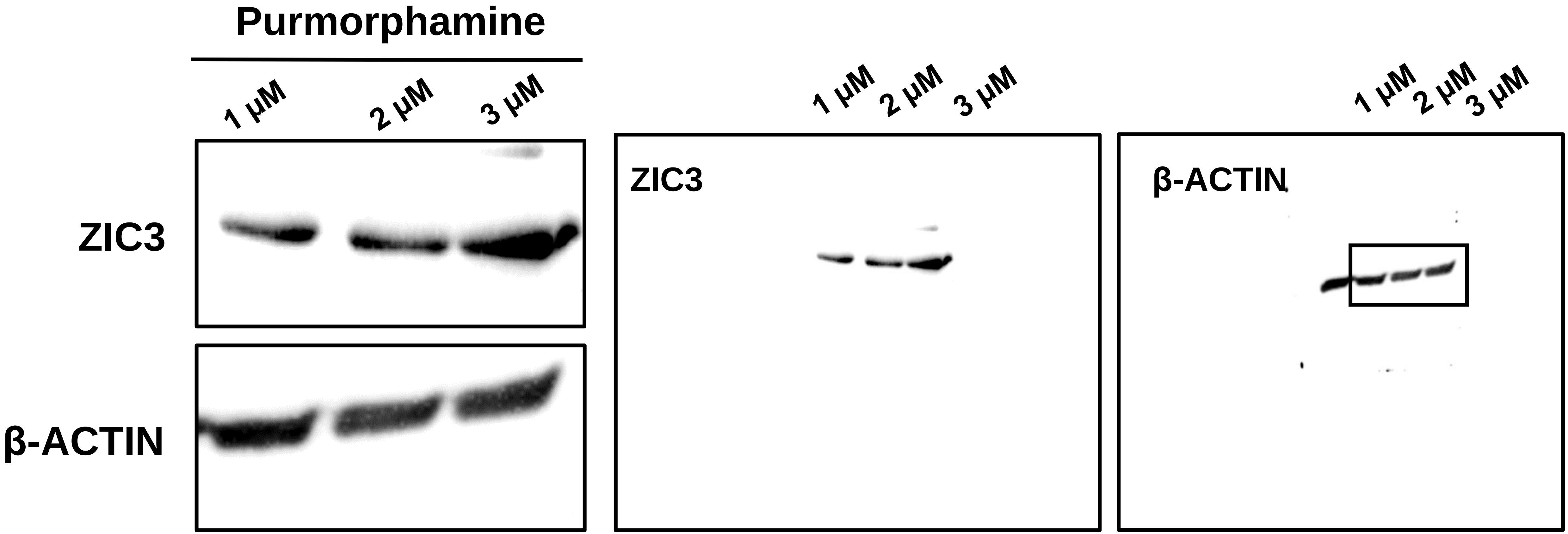

Purmorphamine
1 µM
2 µM
3 µM
ZIC3
β-ACTIN
1 µM
2 µM
3 µM
ZIC3
1 µM
2 µM
3 µM
β-ACTIN

Supplementary Figure S4.c

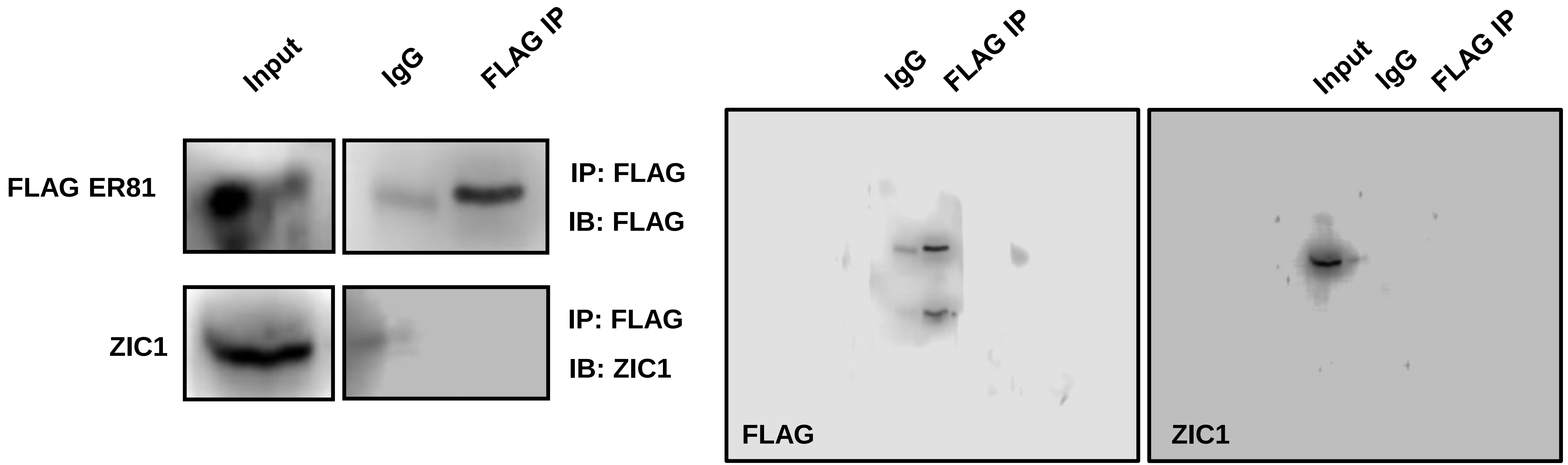

Figure 5.d (i)

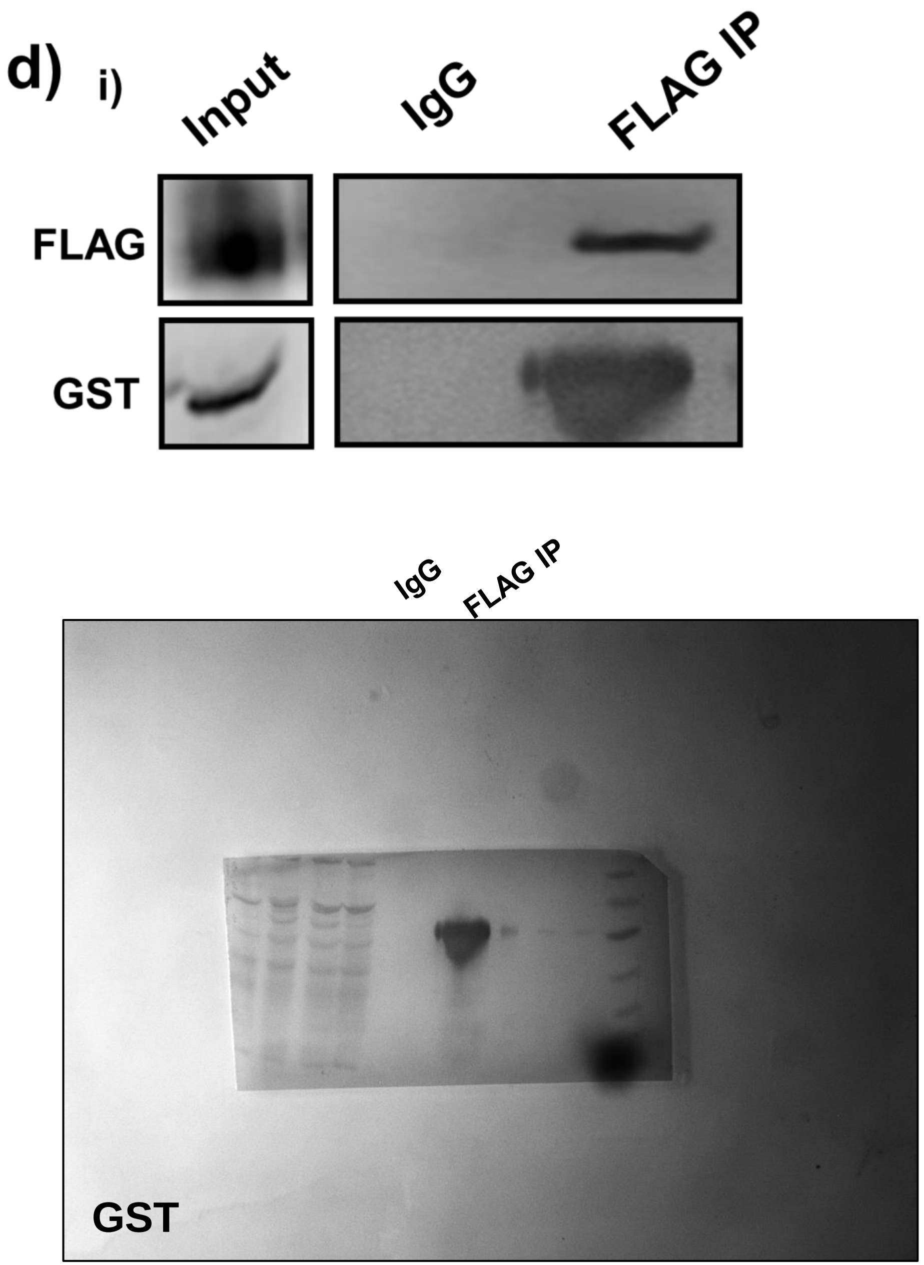

Figure 5.d (ii)

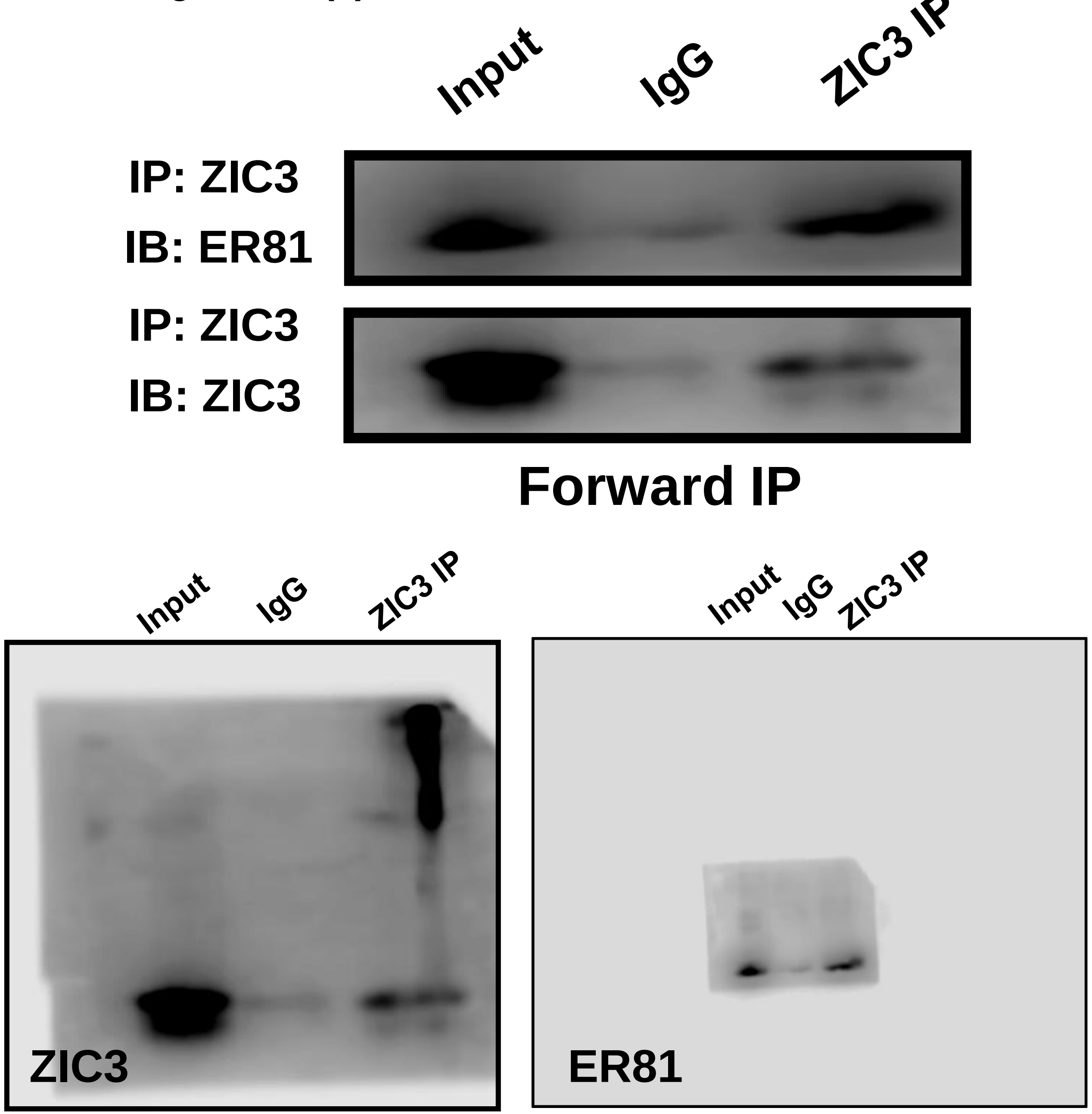

Figure 5.d (ii)

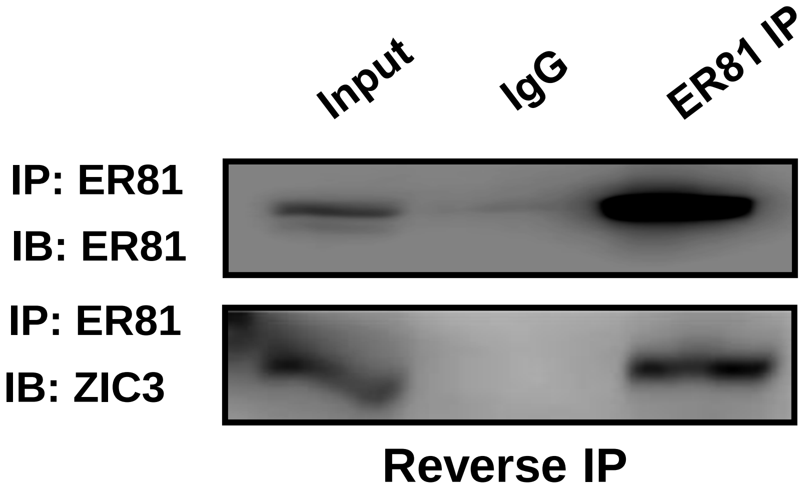
Input
IgG
ER81 IP
IP: ER81
IB: ER81
IP: ER81
IB: ZIC3
Reverse IP

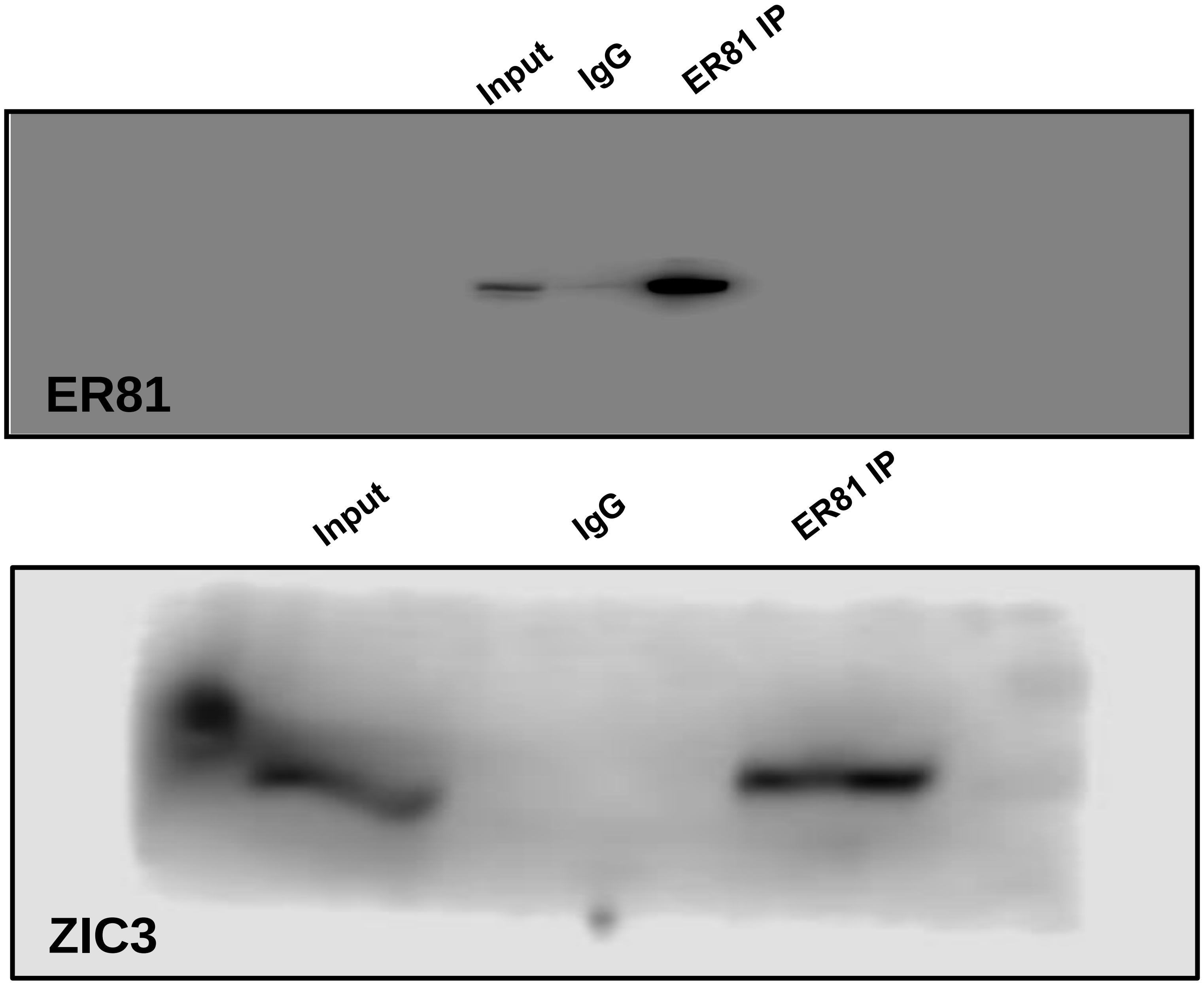
Input
IgG
ER81 IP
ER81
Input
IgG
ER81 IP
ZIC3

Figure 5.e (i)

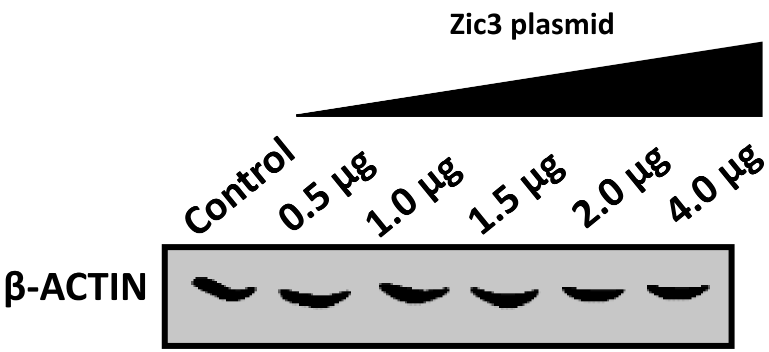

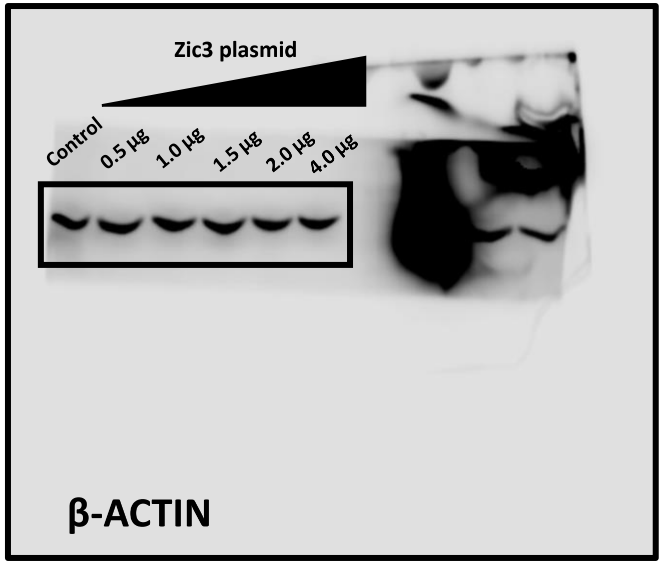

Figure 5.e (ii)

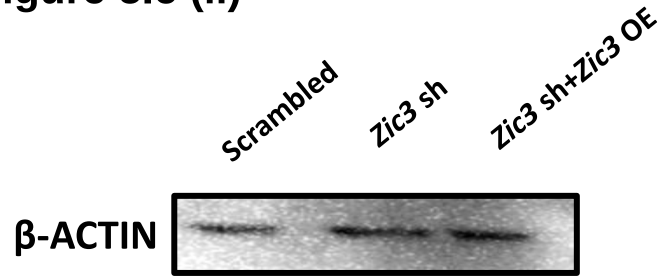

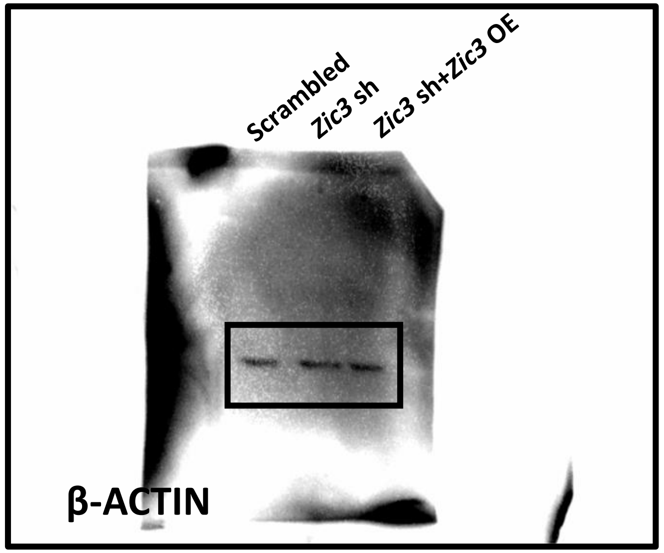

Figure 5.e (iii)

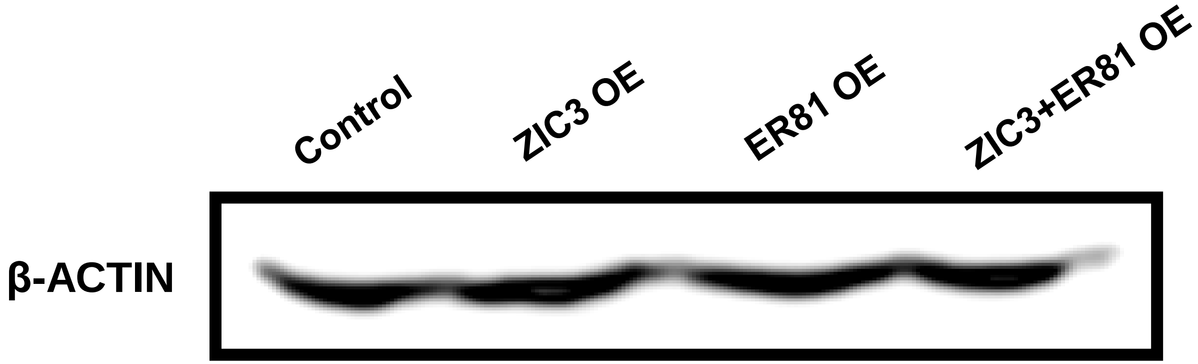

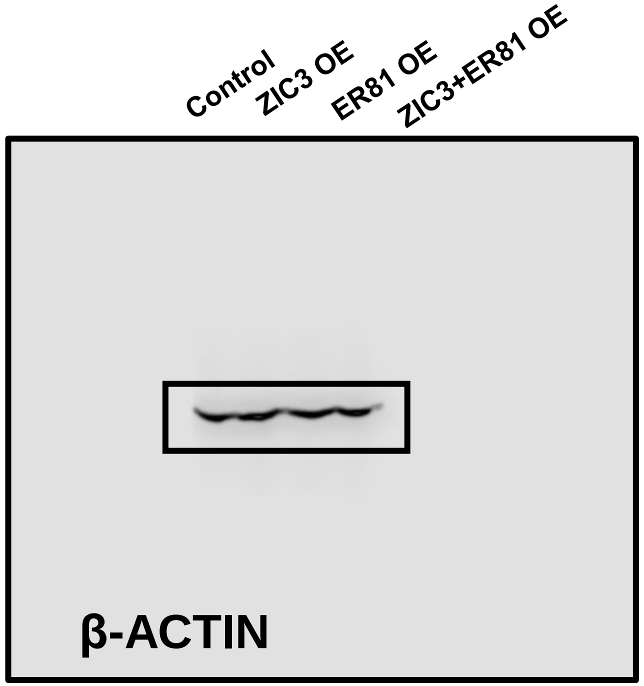

Figure 5.f (i)

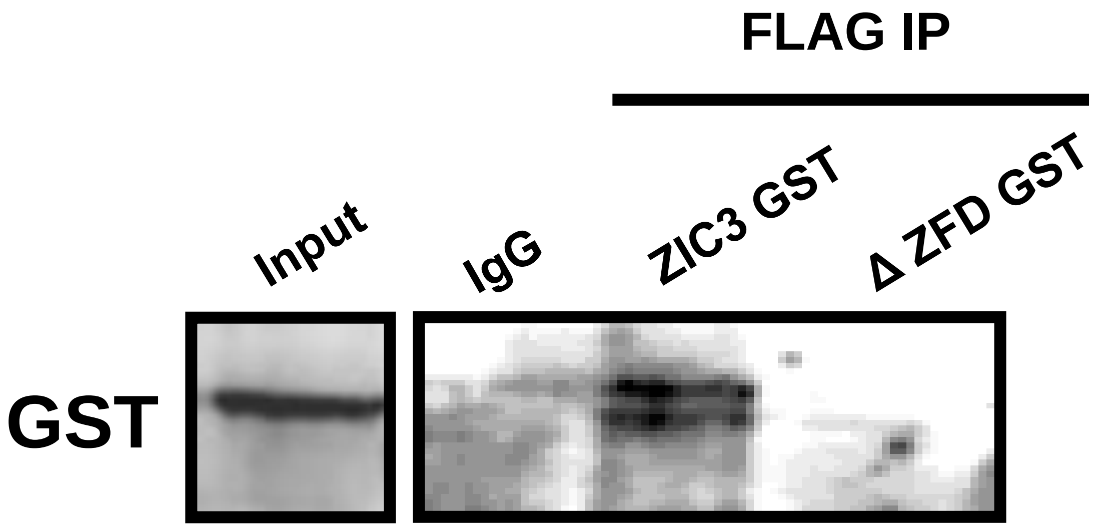
FLAG IP
Input
IgG
ZIC3 GST
Δ ZFD GST
GST

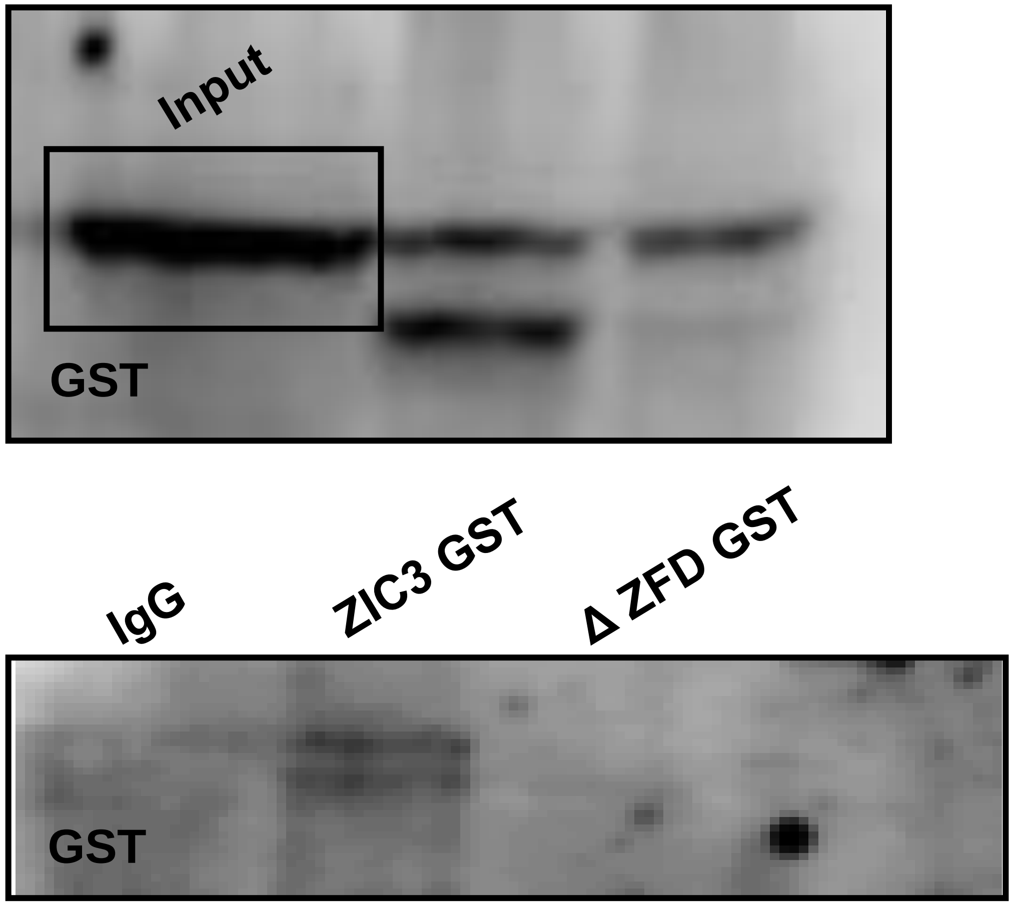
Input
GST
IgG
ZIC3 GST
Δ ZFD GST
GST

Figure 5g

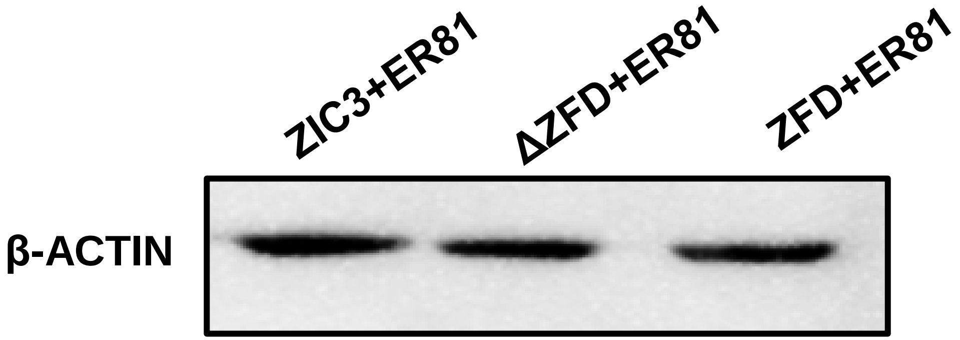
ZIC3+ER81
ΔZFD+ER81
ZFD+ER81
β-ACTIN

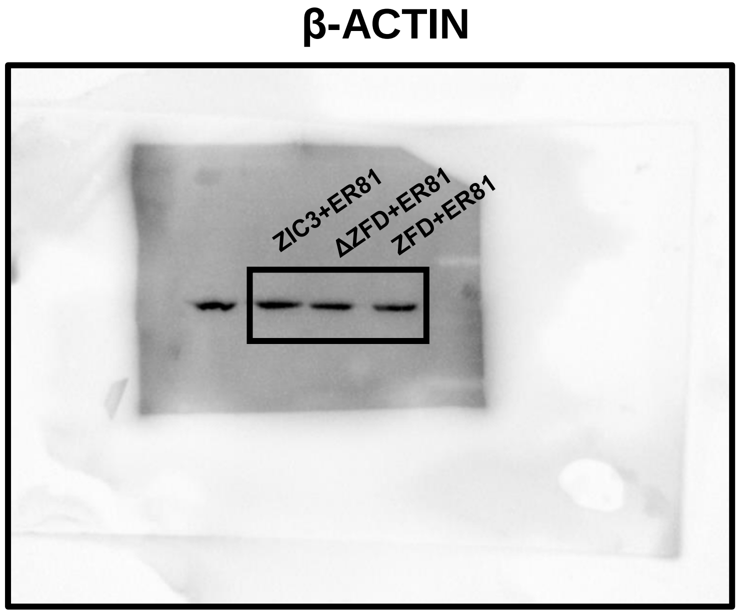
β-ACTIN
ZIC3+ER81
ΔZFD+ER81
ZFD+ER81
